# Temporal Single-Nucleus Transcriptomics Defines PPARα/CPT1A-Associated Metabolic Reprogramming During Proximal Tubular Injury and Repair

**DOI:** 10.3390/biomedicines14102317

**Published:** 2026-10-12

**Authors:** Tao Xu, Peihan Wang, Qi Liu, Jiayi Tao

**Affiliations:** 1 Department of Urology, Huanggang Central Hospital, Affiliated Huanggang Hospital, Hubei University of Science and Technology, Huanggang 438000, China; xutaodsad@whu.edu.cn; 2 Department of Urology, Renmin Hospital, Wuhan University, Wuhan 430000, China; peihanurology@whu.edu.cn

**Keywords:** acute kidney injury, proximal tubule, metabolic reprogramming, mitochondrial dysfunction, fatty acid oxidation, PPARα, CPT1A, cell-state remodeling

## Abstract

**Background:** Metabolic reprogramming and mitochondrial dysfunction are central features of acute kidney injury (AKI), yet their temporal relationship with proximal tubular cell-state remodeling remains incompletely understood. This study aimed to characterize early metabolic alterations and proximal tubular cell-state changes following ischemia–reperfusion injury (IRI). **Methods:** We integrated temporal single-nucleus RNA sequencing with complementary animal and cell-based experiments. The GSE139107 dataset comprised 79,526 kidney nuclei from 12 biological samples collected at Sham, 4 h, 12 h, and 2 d, including 34,151 proximal tubular nuclei. Analyses included subclustering, sample-level pseudobulk analysis, functional gene-set scoring, trajectory inference, and cell–cell communication analysis. Experimental validation was performed using a male mouse IRI model at 24 h of reperfusion and a tubular epithelial cell hypoxia–reoxygenation model. **Results:** Proximal tubular subclustering identified mature and segment-specific populations alongside stressed, injury-associated, repair-associated, and cycling states. Fatty acid oxidation, oxidative phosphorylation, and tubular transport programs were most strongly suppressed at 12 h, accompanied by reduced *Ppara*, *Cpt1a*, *Sdhb*, and *Slc34a1* expression and increased injury-associated gene expression. Partial recovery of metabolic and transport programs occurred at 2 d, although cellular heterogeneity persisted. Monocle3 and Slingshot inferred transitions from mature proximal tubular cells toward stress-, repair-, cycling-, and injury-associated states, accompanied by declining metabolic and transport signatures. CellChat predicted remodeling of extracellular-matrix, adhesion, growth-factor, and guidance-related signaling. In male mice, IRI reduced PPARα, CPT1A, ATP5A, SDHB, and SLC34A1 levels while increasing KIM-1 and NGAL. Hypoxia–reoxygenation similarly decreased PPARα and CPT1A and increased cleaved caspase-3 in tubular epithelial cells. **Conclusions:** These findings reveal a temporally coordinated association among PPARα/CPT1A-related metabolic reprogramming, mitochondrial impairment, and proximal tubular cell-state remodeling during early ischemic AKI, highlighting the dynamic relationship between metabolic dysfunction and tubular injury responses.

## 1. Introduction

Acute kidney injury (AKI) encompasses a heterogeneous group of clinical conditions in which renal function deteriorates rapidly after ischemic, toxic, inflammatory, infectious, or hemodynamic stress. AKI is associated with prolonged hospitalization, increased short-term mortality, and substantial healthcare burden. Even after apparent functional recovery, affected patients remain at increased risk of recurrent AKI, chronic kidney disease, kidney failure, cardiovascular complications, and long-term mortality [1,2,3,4,5]. The outcome of AKI is determined by the severity of the initial insult and the subsequent balance among tubular epithelial survival, adaptive repair, persistent inflammation, vascular recovery, and stromal activation. Defining the molecular events that connect acute tubular damage with successful or maladaptive repair is therefore important for identifying therapeutic strategies that preserve kidney function and prevent disease progression.

The high energetic cost of solute reabsorption renders proximal tubular cells especially sensitive to interruptions in oxygen delivery during renal ischemia–reperfusion. To sustain polarized transport and brush-border function, differentiated proximal tubular cells maintain a mitochondria-rich metabolic phenotype dominated by fatty acid oxidation and oxidative phosphorylation [6,7,8]. During AKI, impaired oxygen delivery and mitochondrial damage disrupt this metabolic program, resulting in ATP depletion, lipid accumulation, oxidative stress, loss of transport capacity, and increased susceptibility to cell death and maladaptive repair [9]. Renal lipid utilization is coordinated partly through PPARα-dependent transcription, while CPT1A regulates the transfer of long-chain fatty acids into mitochondria for β-oxidation. Experimental preservation of proximal tubular PPARα activity supports FAO, mitochondrial gene expression, renal morphology, and kidney function following ischemic or nephrotoxic injury [10]. Conversely, reduced PPARα activity and defective FAO are associated with intracellular lipid accumulation, energy depletion, tubular dedifferentiation, and tissue damage [11]. Mitochondrial metabolic competence is also supported by PGC-1α, which promotes mitochondrial biogenesis, NAD biosynthesis, and resistance to ischemic stress [12]. These findings identify the PPARα/CPT1A-associated metabolic program as an important component of proximal tubular fitness. However, its temporal behavior across distinct epithelial states during the early injury and repair phases remains insufficiently defined.

Single-cell and single-nucleus transcriptomic technologies have revealed extensive heterogeneity within the injured proximal tubular compartment. Following ischemic injury, differentiated proximal tubular cells may acquire segment-specific stress responses and enter injury-associated, dedifferentiated, proliferative, repair-associated, or failed-repair states [13,14]. Kirita et al. identified an inflammatory and profibrotic failed-repair proximal tubular population and showed that injured epithelial cells participate in dynamic signaling with immune and stromal compartments [15]. Subsequent single-nucleus and lineage-tracing studies further identified Vcam1-positive, Ccl2-positive, cycling, and repair-associated proximal tubular states with distinct temporal distributions and biological outcomes [16,17]. These observations indicate that proximal tubular injury cannot be adequately represented by a single damaged-cell phenotype. Instead, injured cells occupy a continuum of transcriptional states that differ in metabolic competence, transport identity, inflammatory activity, proliferative potential, and capacity for redifferentiation. Tubular epithelial cells also contribute actively to the renal microenvironment through cytokines, chemokines, growth factors, extracellular-matrix signals, and adhesion pathways. Ligand-receptor inference tools, including CellChat, provide a computational means of comparing potential signaling relationships among annotated cell populations. Such predictions, however, depend on transcript abundance and curated interaction databases and therefore require experimental validation [18]. Nevertheless, predicted communication networks remain hypothesis-generating, and the temporal relationship among metabolic reprogramming, proximal tubular state remodeling, and multicellular signaling during early AKI remains incompletely resolved.

To characterize early proximal tubular remodeling, we reanalyzed the GSE139107 mouse kidney dataset at Sham, 4 h, 12 h, and 2 d using whole-kidney annotation, PT subclustering, sample-level pseudobulk analysis, functional scoring, trajectory inference, and CellChat. The temporal patterns were defined from the GSE139107 transcriptomic series. Selected molecular observations were subsequently assessed at a separate 24-h endpoint in a male mouse renal ischemia–reperfusion model and in HK-2 cells exposed to hypoxia–reoxygenation. These experimental analyses were designed to provide complementary protein-, tissue-, and functional-level evidence rather than to reproduce the complete 4 h–12 h–2 day transcriptomic kinetics.

## 2. Materials and Methods

### 2.1. Study Design

The study consisted of two components: secondary analysis of GSE139107 and experimental validation in mouse and cell models. The transcriptomic component included three kidneys at each of four time points, yielding 12 biological samples. The temporal component of the study was derived exclusively from GSE139107. The independent animal experiment used a single 24h reperfusion endpoint and was designed to assess selected molecular and tissue-level findings cross-sectionally rather than to reproduce or validate the complete 4 h, 12 h, and 2 day transcriptomic trajectory. Technical libraries originating from the same kidney were merged under a shared biological-sample identifier. In the animal study, group allocation was randomized, whereas histological assessment and image quantification were performed under blinded conditions.

### 2.2. Public Single-Nucleus RNA Sequencing Dataset and Sample Definition

Single-nucleus RNA sequencing data were obtained from the Gene Expression Omnibus under accession number GSE139107. The original dataset included mouse kidneys collected at Sham and at 4 h, 12 h, 2 d, 14 d, and 42 d after bilateral renal ischemia–reperfusion injury. The present study focused on the acute injury and early repair period represented by Sham, 4 h, 12 h, and 2 d. The analyzed dataset contained 79,526 nuclei from 12 independent kidneys. Biological-sample identifiers were reconstructed from the original metadata and cell barcodes. Technical sequencing libraries carrying different batch suffixes but originating from the same kidney were assigned to a single biological sample. The resulting sample identifiers were Sham1–Sham3, 4h1–4h3, 12h1–12h3, and 2d1–2d3. A complete mapping between the original sequencing-library identifiers and the reconstructed biological samples is provided in Supplementary Table S1. All statistical comparisons between experimental time points were performed at the biological-kidney level. Individual nuclei and technical sequencing libraries were not treated as independent biological replicates. No additional ethical approval was required for the secondary analysis of the publicly available dataset.

### 2.3. Single-Nucleus RNA Sequencing Data Processing

Single-nucleus transcriptomic analyses were performed in R v4.5.1 using Seurat v5.5.0 and SeuratObject v5.2.0 [19]. Raw RNA count matrices and associated metadata were retained throughout the analysis. Seurat v5 RNA layers were joined using JoinLayers (version 5.2.1) before analyses requiring unified counts or normalized-expression matrices were performed. Nuclei were retained when 500–6000 genes and 1000–30,000 RNA counts were detected and mitochondrial transcripts accounted for less than 5% of total counts. Doublets were evaluated separately for each biological sample using DoubletFinder v2.0.6. The optimal pK value was selected by parameter sweeping, pN was fixed at 0.25, and the expected 7.5% doublet rate was adjusted for homotypic events. After doublet removal, counts were normalized with LogNormalize (version 5.2.1) using a scale factor of 10,000. The 3000 most variable genes were used for scaling and principal component analysis. A shared nearest-neighbor graph based on the first 30 components was clustered with the Louvain algorithm at a resolution of 0.5, followed by UMAP visualization.

### 2.4. Whole-Kidney Cell Annotation

Whole-kidney clusters were independently annotated using established lineage markers and were subsequently cross-checked against the annotations reported in the original dataset. Major populations included proximal tubular cells, injury- or repair-associated proximal tubular cells, collecting duct cells, distal convoluted and connecting tubular cells, thick ascending limb cells, cycling epithelial cells, urothelium-like cells, endothelial cells, stromal cells, macrophage or monocyte cells, and podocytes. Proximal tubular cells were identified by expression of *Slc34a1*, *Lrp2*, *Cubn*, and *Slc5a2*. Thick ascending limb cells expressed *Umod* and *Slc12a1*, whereas collecting duct cells expressed *Aqp2* and *Fxyd4*. Distal tubular populations were supported by *Pvalb*. Endothelial cells expressed *Pecam1*, *Emcn*, and *Kdr*; stromal cells expressed *Col1a1*, *Col1a2*, and *Dcn*; macrophage or monocyte populations expressed *Lyz2*, *Adgre1*, and *Csf1r*; and podocytes expressed *Nphs1* and *Nphs2*. Cluster identity was assigned only when the overall marker profile was biologically coherent. Clusters dominated by stress, cell-cycle, or injury-associated genes were distinguished from mature nephron populations using combined lineage and functional-marker expression.

### 2.5. Proximal Tubular Extraction, Subclustering, and State Annotation

Nuclei assigned to the proximal tubular lineage were extracted from the whole-kidney object. The final proximal tubular dataset contained 34,151 nuclei. The RNA assay was independently normalized, highly variable genes were identified, and principal component analysis, neighbor-graph construction, clustering, and UMAP visualization were repeated using the procedures described above. Proximal tubular nuclei were classified into seven transcriptional states: mature PT, PT_S1_S2, PT_S3, stressed PT, injury-associated PT, repair-associated PT, and cycling PT. Mature and segment-specific states were defined by combinations of *Slc34a1*, *Lrp2*, *Cubn*, *Slc5a2*, *Aqp1*, and related solute-transport genes. Metabolically competent states were supported by expression of *Ppara*, *Ppargc1a*, *Cpt1a*, *Cpt2*, *Acox1*, *Acadl*, *Acadm*, *Atp5a1*, and *Sdhb*. Injury-associated PT cells showed increased expression of *Havcr1*, *Lcn2*, *Krt8*, *Krt18*, *Vcam1*, and *Spp1*. Repair-associated PT cells retained partial proximal tubular identity while expressing epithelial activation, dedifferentiation, and repair-related genes, including *Sox9*, *Vim*, and *Vcam1*. The designation “repair-associated” refers to a transcriptional state associated with the injury-response and repair process and does not imply completed structural or functional recovery. Cycling PT cells were identified by *Mki67*, *Top2a*, *Cdk1*, and other cell-cycle genes. Marker genes were identified using the Wilcoxon rank-sum test implemented in FindAllMarkers. Genes were required to be positively enriched, detected in at least 25% of cells in the corresponding population, and have an average log2 fold change greater than 0.25. *p* values were adjusted using the Benjamini–Hochberg procedure, with an adjusted *p* value below 0.05 considered significant. Cell-level marker testing was used for cluster annotation and descriptive characterization rather than for condition-level statistical inference.

### 2.6. Analysis of Renal Cell and Proximal Tubular State Composition

Cell-type and proximal tubular state abundances were calculated independently for each biological kidney. For whole-kidney analysis, the number of nuclei assigned to each cell type was divided by the total number of nuclei recovered from the corresponding kidney. For proximal tubular analysis, the number of nuclei assigned to each PT state was divided by the total number of proximal tubular nuclei in that kidney. Technical libraries from the same kidney were combined before calculating proportions. Because cell-population proportions are compositional and sum to one within each sample, overall time-dependent differences were evaluated using Dirichlet-multinomial regression with time point included as the explanatory variable [20]. Pairwise contrasts between each injury time point and Sham were corrected using the Benjamini–Hochberg method. Sample-level proportions were displayed as stacked bar charts and individual-point plots.

### 2.7. Functional Gene-Set Scoring

Functional programs were quantified using AddModuleScore in Seurat with identical scoring parameters across all samples and time points. All module scores were recalculated from the final proximal tubular object to ensure methodological consistency. The fatty acid oxidation module included *Ppara*, *Ppargc1a*, *Cpt1a*, *Cpt2*, *Acox1*, *Acadl*, *Acadm*, *Acads*, *Hadha*, *Hadhb*, *Eci1*, *Etfa*, and *Etfb*. The oxidative phosphorylation module included representative genes from respiratory-chain complexes and ATP synthase: *Ndufa1*, *Ndufa2*, *Ndufb3*, *Ndufs1*, *Ndufs2*, *Sdha*, *Sdhb*, *Uqcrc1*, *Uqcrc2*, *Cox4i1*, *Cox5a*, *Atp5a1*, and *Atp5b*. The proximal tubular transport module included *Slc34a1*, *Slc5a2*, *Lrp2*, *Cubn*, *Aqp1*, *Slc22a6*, and *Slc22a8*. The tubular injury module included *Havcr1*, *Lcn2*, *Krt8*, *Krt18*, *Vcam1*, and *Spp1*.

Cell-level scores were projected onto the proximal tubular UMAP for visualization. For condition-level comparisons, module scores were averaged across proximal tubular nuclei within each biological kidney, yielding 12 independent sample-level observations. Statistical testing was conducted using these sample-level values. Because genes used for PT-state annotation overlapped with several functional programs, including metabolic, transport, and injury-related modules, module-score differences among transcriptionally defined PT states were interpreted descriptively and were not considered independent validation of state identity. For condition-level inference, module scores were averaged within each biological kidney, and temporal comparisons across biological kidneys were considered the primary module-score analysis. To assess the influence of annotation-marker overlap, sensitivity analyses were additionally performed after excluding a predefined set of 40 annotation/review genes from the relevant external gene sets and corresponding control or ranking universes. These marker-excluded analyses were interpreted as robustness analyses rather than independent biological validation.

### 2.8. Sample-Level Pseudobulk Expression Analysis

Raw RNA counts from proximal tubular nuclei were aggregated by the biological kidney to generate 12 pseudobulk expression profiles. For each gene, counts were summed across all proximal tubular nuclei from the corresponding sample. Pseudobulk differential expression analysis was performed using edgeR v4.8.2 and limma v3.66.0 within Bioconductor v3.22 [21,22]. A DGEList object was constructed from the aggregated count matrix, and genes with low expression were filtered using filterByExpr, with experimental time points included in the design matrix. Library-size differences were normalized using the trimmed mean of M values method implemented in calcNormFactors. Normalized counts were transformed using the voom method to model the mean-variance relationship, followed by linear modeling with lmFit. Prespecified contrasts were fitted using contrasts.fit, and moderated statistics were calculated using empirical Bayes shrinkage with eBayes. The design matrix contained four groups: Sham, 4 h, 12 h, and 2 d. The prespecified contrasts were 4 h versus Sham, 12 h versus Sham, 2 d versus Sham, 12 h versus 4 h, and 2 d versus 12 h. For each tested gene, we reported the estimated log2 fold change, pointwise 95% confidence interval, exact numerical *p* value, and Benjamini–Hochberg-adjusted *p* value. Complete results are provided in Supplementary Data S2. To examine the robustness of the kidney-level estimates, we performed systematic leave-one-kidney-out refitting without selecting samples for exclusion. We additionally performed within-published-PT-subgroup pseudobulk analyses for subgroup-by-kidney strata containing at least 50 nuclei and three eligible kidneys on both sides of a comparison. CAMERA was used as a correlation-aware pathway sensitivity analysis. Complete sensitivity results are provided in Supplementary Figure S5 and Supplementary Data S5. *p* values were corrected using the Benjamini–Hochberg method. Genes with an adjusted *p* value below 0.05 were considered differentially expressed. Genes that did not pass the expression filter were not assigned inferential *p* values. If biologically relevant low-expression genes were displayed, their expression patterns were presented descriptively and clearly distinguished from statistically tested genes. Expression values used in time-course plots were reported as log2 counts per million. For heatmap visualization, expression was standardized separately for each gene and truncated to a range of −2 to 2. The biological kidney, rather than the individual nucleus, served as the statistical unit throughout the pseudobulk analysis [23].

### 2.9. Monocle3 Trajectory Inference

Proximal tubular cell-state relationships were reconstructed using monocle3 version 1.4.25 [24]. The Seurat proximal tubular object was converted into a cell_data_set object, with raw RNA counts, cell metadata, proximal tubular state annotations, and UMAP coordinates transferred after matching shared cell barcodes. A principal graph was learned in the existing UMAP embedding using the learn_graph function with default parameters. Trajectory plots were colored by PT state, pseudotime, experimental time point, and biological sample. Because trajectory direction depends partly on root selection, the inferred ordering was interpreted as a computational representation consistent with transition from mature toward stress-, injury-, and repair-associated transcriptional states. It was not interpreted as direct lineage tracing. For sample-level comparison, the median pseudotime of all proximal tubular nuclei was calculated separately for each biological kidney. Time-dependent differences in median pseudotime were evaluated using a sample-level linear model, followed by multiplicity-adjusted contrasts against Sham.

### 2.10. Identification of Pseudotime-Associated Genes

Genes associated with the Monocle3 principal graph were identified using graph_test, with the principal graph specified as the neighborhood structure. Genes with a false discovery rate below 0.01 and a positive Moran’s I statistic were considered pseudotime-associated. The 48 highest-ranking genes were selected according to adjusted significance and Moran’s I. Cells were ordered by pseudotime and divided into 45 approximately equal-sized bins. Normalized expression was averaged within each bin and standardized separately for each gene. Values were truncated between −2 and 2 for visualization. Dynamic genes were divided into four expression modules using k-means clustering with 30 random initializations. Within each module, genes were ordered according to the pseudotime bin at which their standardized expression reached its maximum. The resulting modules were interpreted according to enrichment for differentiated transport, metabolic, stress-response, cell-cycle, injury, and repair-associated programs.

### 2.11. Sample-Aware Modeling of Functional Programs Along Pseudotime

To evaluate changes in functional programs along pseudotime while accounting for biological replication, proximal tubular cells were divided into 20 equal-width pseudotime intervals separately within each kidney. Mean fatty acid oxidation, oxidative phosphorylation, proximal tubular transport, and injury scores were calculated for each sample-by-bin combination. Bins containing fewer than 20 cells were excluded to reduce instability caused by sparse cell representation. Generalized additive models were fitted using the mgcv package version 1.9-4. For each functional program, the mean sample-by-bin score was modeled as a smooth function of scaled pseudotime using a penalized thin-plate regression spline with a maximum basis dimension of six. Biological sample was included as a random intercept to account for correlations among pseudotime bins derived from the same kidney. Models assumed a Gaussian error distribution with an identity link, and smoothing parameters were estimated using restricted maximum likelihood. Predicted values and corresponding 95% confidence intervals were calculated at 120 evenly spaced positions across the scaled pseudotime range. Observed sample-level bin means were superimposed on the fitted curves for visualization.

### 2.12. Slingshot Trajectory Analysis

Slingshot v2.18.0 was used as a complementary trajectory-inference method [25]. A SingleCellExperiment object was constructed using the proximal tubular UMAP coordinates and PT-state annotations. Mature PT was specified as the starting cluster based on its enrichment in Sham kidneys and expression of differentiated proximal tubular markers. Lineage curves were fitted using 200 approximation points. For cells assigned to multiple lineages, a weighted pseudotime value was calculated from the lineage-specific pseudotime values and the corresponding curve weights. Slingshot pseudotime values were rescaled to a range of 0 to 1 for comparison with Monocle3 pseudotime. Agreement between Monocle3 and Slingshot was evaluated descriptively using Spearman correlation of cell-level pseudotime values and by comparing the median pseudotime ordering of the annotated PT states. Spearman’s correlation coefficient and the corresponding two-sided *p* value were reported. Because both approaches were applied to the same cells and low-dimensional representation, Slingshot was considered a complementary robustness analysis rather than an independent biological validation.

### 2.13. Exploratory Cell–Cell Communication Analysis

Intercellular communication was explored using CellChat v2.1.2 and the CellChatDB v2 mouse ligand-receptor interaction database (CellChatDB.mouse). CellChat estimates communication probabilities by integrating the expression of ligands, receptors, multimeric receptor complexes, agonists, antagonists, and signaling cofactors within a mass-action framework. For continuity with the original analysis, the comparison between Sham and pooled early-AKI samples was retained as a descriptive overview of the global communication landscape. The pooled early-AKI group included nuclei collected at 4 h, 12 h, and 2 day. However, because these time points represent distinct biological stages, additional time-specific CellChat analyses were performed separately for Sham, 4 h, 12 h, and 2 day to resolve temporal communication dynamics. To improve comparability across time points, a common set of cell populations and sender–receiver relationships was defined before network comparison. Cell populations were required to contain at least 10 nuclei in at least two biological kidneys at every time point, yielding 15 common cell types and 213 comparable sender–receiver combinations. Within each time point and cell type, eligible kidneys contributed equal numbers of nuclei, with sampling capped at 300 nuclei per kidney/cell type, to reduce disproportionate influence from kidneys with larger cell recoveries. In addition to the four time-point-level networks, 12 individual-kidney CellChat models were constructed to evaluate the reproducibility of predicted ligand-receptor relationships across biological replicates. A candidate relationship was considered evaluable only when both the sender and receiver populations were present in the corresponding kidney. Kidney-level support was reported as the number of kidneys in which the relationship was detected divided by the number of evaluable kidneys. This support measure was used descriptively and was not interpreted as a between-condition significance test. Communication probabilities were calculated using the trimean estimator. Predicted interaction number, aggregate communication strength, pathway-level information flow, and PT-centered ligand-receptor candidates were summarized for each time point. The 12 h versus Sham comparison was emphasized because 12 h represented the period of strongest injury-associated transcriptional disturbance in the present dataset. Complete time-specific networks, individual-kidney support summaries, and ligand-receptor expression data are provided in Supplementary Figure S8 and Supplementary Data S8–S11. Because CellChat is based on transcript abundance and curated ligand-receptor relationships, these results were interpreted as predicted communication patterns. Neither pooled nor time-specific CellChat inference establishes ligand secretion, receptor activation, spatial contact, or downstream functional signaling.

### 2.14. Animals and Ethical Approval

Male C57BL/6J mice aged 8–10 weeks and weighing 20–25 g were obtained from the Laboratory Animal Center of Renmin Hospital of Wuhan University (Wuhan, China). The present animal experiment was designed as a focused proof-of-concept validation of selected molecular and tissue-level findings and was conducted using a single-sex male cohort. The study was not designed to evaluate sex-dependent differences in renal ischemia–reperfusion injury or metabolic responses. Accordingly, the in vivo findings are interpreted as being specific to male mice, and extrapolation to female animals requires further validation. Animals were allocated to the Sham or AKI group using a computer-generated random sequence, with six mice in each group. Investigators responsible for histological scoring, image analysis, and Western blot quantification were blinded to group allocation. The experimental protocol was reviewed and approved by the Animal Ethics Committee of Renmin Hospital of Wuhan University (approval no. WDRM20200308A). All animal procedures were conducted in accordance with institutional guidelines for the care and use of laboratory animals and were reported in accordance with the ARRIVE 2.0 guidelines.

### 2.15. Renal Ischemia–Reperfusion Injury

Anesthesia was induced by intraperitoneal administration of sodium pentobarbital at 50 mg/kg and was confirmed by the absence of the pedal withdrawal response. Mice were positioned on a heated surgical platform, and core body temperature was maintained at 36.5–37.0 °C throughout the procedure. Following a midline laparotomy, both renal pedicles were carefully isolated and occluded using atraumatic microvascular clamps for 30 min. Successful ischemia was confirmed by visible darkening of the kidneys, and restoration of normal renal color following clamp removal was used to confirm reperfusion. Sham-operated mice underwent identical surgical exposure of the renal pedicles without vascular occlusion. For perioperative analgesia, buprenorphine was administered subcutaneously at 0.05 mg/kg before surgery and subsequently at 12-h intervals until the experimental endpoint. Immediately after surgery, mice received 0.5 mL of prewarmed sterile saline and were allowed to recover under external heating. At 24 h after reperfusion, mice were euthanized by an intraperitoneal overdose of sodium pentobarbital at 150 mg/kg. Cardiac blood was collected after deep anesthesia had been established, and cervical dislocation was subsequently performed as a secondary physical method to confirm death. Kidney tissues were then rapidly harvested for subsequent analyses.

### 2.16. Assessment of Renal Function

Blood was collected by cardiac puncture under deep anesthesia immediately before euthanasia and allowed to clot at room temperature for 30 min. Samples were centrifuged at 3000× *g* for 10 min at 4 °C. The resulting serum was carefully collected, divided into aliquots, and stored at −80 °C until analysis. Serum creatinine was measured using a Creatinine Assay Kit (Abcam, Cambridge, UK; catalog no. ab65340), and blood urea nitrogen was determined using a Urea Assay Kit (Abcam, Cambridge, UK; catalog no. ab83362). Both kits are suitable for quantitative analysis of creatinine or urea in mouse serum. Assays were performed in duplicate according to the manufacturers’ instructions, and the mean of the technical replicates was used for statistical analysis. Each biological replicate represented serum collected from one independent mouse.

### 2.17. Histological Examination and Tubular Injury Scoring

Kidney tissues were fixed in 4% paraformaldehyde for 24 h, dehydrated through a graded ethanol series, embedded in paraffin, and sectioned at a thickness of 4 μm. Sections were stained with hematoxylin and eosin according to standard procedures. Tubular injury was evaluated in the renal cortex and the outer stripe of the outer medulla based on tubular dilation, epithelial flattening, brush-border loss, cast formation, luminal debris, cellular detachment, and necrosis. Injury severity was graded using a semiquantitative scale from 0 to 5, where 0 indicated no detectable injury, 1 indicated involvement of less than 10% of tubules, 2 indicated involvement of 10% to 25%, 3 indicated involvement of 26% to 50%, 4 indicated involvement of 51% to 75%, and 5 indicated involvement of more than 75% of tubules. Ten randomly selected, non-overlapping fields were examined per kidney at 200× magnification using a systematic random sampling approach. Two investigators blinded to group allocation independently performed the scoring. The mean score across all fields and both observers was calculated for each mouse and used as one biological replicate.

### 2.18. Immunohistochemistry

After deparaffinization and rehydration, sections underwent citrate-based antigen retrieval at pH 6.0 for 15 min. Peroxidase activity was quenched with 3% hydrogen peroxide, and nonspecific binding was blocked with 5% bovine serum albumin. KIM-1 and NGAL primary antibodies were applied overnight at 4 °C, followed by HRP-conjugated secondary antibodies and DAB development. Images were collected under fixed acquisition conditions. Mean optical density was calculated from 10 cortical and outer-medullary fields per kidney using a uniform ImageJ threshold, after which field values were averaged for each animal.

### 2.19. Immunofluorescence Staining

Kidney sections were deparaffinized in xylene, rehydrated through a graded ethanol series, and subjected to heat-induced antigen retrieval in 10 mM citrate buffer at pH 6.0 for 15 min. Sections were permeabilized with 0.3% Triton X-100 for 10 min and blocked with 5% bovine serum albumin for 1 h at room temperature. The sections were then incubated overnight at 4 °C with Lotus tetragonolobus lectin to identify proximal tubular brush borders and a primary antibody against SLC34A1. Reagent manufacturers, catalog numbers, and working dilutions are provided in Supplementary Table S2. After washing with phosphate-buffered saline, sections were incubated with the appropriate fluorescent secondary antibodies for 1 h at room temperature in the dark, followed by nuclear counterstaining with DAPI for 5 min. Images were acquired using an Olympus FV3000 laser-scanning confocal microscope (Olympus, Tokyo, Japan) under identical laser power, exposure time, gain, and offset settings for all experimental groups. Ten randomly selected, non-overlapping fields were evaluated per kidney at 400× magnification. LTL-positive proximal tubular regions were segmented using a uniform fluorescence-intensity threshold, and the mean fluorescence intensity of SLC34A1 within the LTL-positive regions was quantified using ImageJ version 1.54 t. The same segmentation and measurement parameters were applied to all images within the same experiment. Field-level measurements were averaged to obtain one value per mouse, which was treated as an independent biological replicate.

### 2.20. Cell Culture and Hypoxia–Reoxygenation Treatment

HK-2 human proximal tubular epithelial cells were obtained from the American Type Culture Collection (ATCC; CRL-2190; RRID). Cells were maintained in DMEM/F-12 supplemented with 10% fetal bovine serum and 1% penicillin–streptomycin at 37 °C in a humidified atmosphere containing 5% CO_2_. Cells between passages 3 and 10 were used for all experiments. Because the cells were obtained directly from the ATCC and used at low passage numbers, no additional STR-based authentication was performed in our laboratory. Cultures were routinely screened for mycoplasma contamination using a PCR-based mycoplasma detection assay before experiments, and only mycoplasma-negative cultures were used. For hypoxia–reoxygenation experiments, HK-2 cells were seeded at 2 × 10^5^ cells per well and subjected to treatment when they reached approximately 70–80% confluence. Cells were transferred to serum-free low-glucose medium and incubated under hypoxic conditions consisting of 1% O_2_, 5% CO_2_, and 94% N_2_ at 37 °C for 24 h. The hypoxic medium was then replaced with the complete culture medium, and cells were reoxygenated for 6 h under normoxic conditions at 37 °C in 5% CO_2_ and atmospheric oxygen. Control cells were maintained under normoxic conditions for the corresponding experimental period.

### 2.21. PPARα Agonist Treatment and CPT1A Knockdown

PPARα agonist treatment and CPT1A knockdown. For functional intervention experiments, HK-2 cells were transfected with a small interfering RNA targeting human CPT1A (siCPT1A) or a non-targeting negative-control siRNA (siNC). The siCPT1A sequences were 5′-CCAUGAAGCUCUUAGACAA-3′ (sense) and 5′-UGUCUAAGAGCUUCAUGG-3′ (antisense). siRNA was transfected at a final concentration of 50 nM using Lipofectamine 3000 (Invitrogen, Thermo Fisher Scientific, Carlsbad, CA, USA) in Opti-MEM according to the manufacturer’s instructions. Cells were approximately 50–70% confluent at the time of transfection. After 6 h, the transfection medium was replaced with fresh complete medium, and subsequent treatments were initiated 24 h after transfection. WY-14643 (Cayman Chemical, Ann Arbor, MI, USA; Item No. 70730), a pharmacological PPARα agonist, was dissolved in DMSO to prepare a stock solution and used at a final concentration of 50 μM. The final concentration of DMSO was maintained at ≤0.1% in all groups. Cells were pretreated with WY-14643 for 1 h before hypoxia and the compound was maintained during the subsequent hypoxia/reoxygenation period. Vehicle-treated groups received an equivalent concentration of DMSO. HK-2 cells were assigned to four groups: (1) control + vehicle + siNC; (2) H/R + vehicle + siNC; (3) H/R + WY-14643 + siNC; and (4) H/R + WY-14643 + siCPT1A. Hypoxia was induced for 24 h under 1% O_2_, 5% CO_2_, and 94% N_2_, followed by 6 h of reoxygenation under normoxic conditions. CPT1A knockdown was confirmed by Western blotting. Cells and culture supernatants were subsequently collected for Western blotting, TMRE staining, ATP measurement, and LDH-release assays.

### 2.22. Western Blotting

Protein was extracted from kidney tissue and HK-2 cells using inhibitor-supplemented RIPA buffer. Tissue lysates were cleared at 12,000× *g* for 15 min at 4 °C, and concentrations were determined by BCA assay. Thirty micrograms of protein were resolved on 8–12% SDS-PAGE gels and transferred to PVDF membranes. After blocking, membranes were incubated with the primary antibodies listed in Supplementary Table S2, followed by HRP-conjugated secondary antibodies. Chemiluminescent signals were recorded using a ChemiDoc MP system and quantified in ImageJ. Target signals were normalized to β-actin, and a pooled reference lysate was used when samples were distributed across different membranes.

### 2.23. TMRE Staining

Mitochondrial membrane potential was evaluated using tetramethylrhodamine ethyl ester (TMRE) staining. After the indicated treatments, HK-2 cells were incubated with TMRE staining solution at 37 °C in the dark according to the manufacturer’s instructions. Cells were subsequently washed to remove excess dye and counterstained with DAPI for nuclear visualization. Fluorescence images were acquired using a fluorescence microscope under identical imaging conditions for all groups. TMRE fluorescence intensity was quantified using ImageJ software (version 1.54p; National Institutes of Health, Bethesda, MD, USA), and the mean fluorescence intensity was normalized to that of the control group.

### 2.24. ATP Assay

Intracellular ATP levels were determined using a commercial ATP assay kit according to the manufacturer’s instructions. Following the indicated treatments, HK-2 cells were collected and lysed, and the resulting cell lysates were used for ATP measurement. Luminescence signals were detected using a microplate reader. ATP levels were calculated from the corresponding signal intensities and expressed relative to the control group, which was set to 1.

### 2.25. LDH Assay

Cellular injury was further assessed by measuring lactate dehydrogenase (LDH) release into the culture medium using a commercial LDH assay kit. After the indicated treatments, cell culture supernatants were collected and processed according to the manufacturer’s instructions. Absorbance was measured using a microplate reader, and LDH release was calculated based on the corresponding optical density values. The results were normalized to the control group and expressed as relative LDH release.

### 2.26. Image and Blot Integrity

All histological and fluorescence images within the same experiment were acquired using identical imaging parameters. Adjustments to brightness, contrast, or color balance, when necessary, were applied uniformly to the entire image and consistently across all comparison groups. No selective modification, removal, relocation, or enhancement of individual image regions or biological features was performed. Western blot images were cropped solely for figure presentation. Uncropped membrane images, molecular-weight markers, exposure information, and source densitometry data are provided in the Supplementary Materials. Any rearrangement of lanes or assembly of images obtained from different membranes is indicated by dividing lines and described in the corresponding figure legends.

### 2.27. Statistical Analysis

Statistical analyses used biological samples as the unit of replication. Animal data are shown as mean ± SD, and technical replicates or multiple imaging fields were averaged before testing. Two-group comparisons used an unpaired two-sided Student’s *t* test when normality and equal-variance assumptions were met, Welch’s test for unequal variances, and the Mann–Whitney U test otherwise. Time-course transcriptomic measures were assessed with sample-level linear or rank-based models, followed by Benjamini–Hochberg correction for comparisons with Sham. Differential expression was evaluated using limma empirical Bayes statistics with false-discovery-rate adjustment. Correlations were tested with Pearson or Spearman methods as appropriate. Statistical significance was set at *p* or adjusted *p* < 0.05. Because only three independent biological kidneys were available per time point, distributional assumptions could not be robustly evaluated. Accordingly, condition-level transcriptomic results were interpreted using the biological kidney as the inferential unit, with emphasis on raw kidney-level values, effect estimates, pointwise 95% confidence intervals, exact numerical *p* values, and multiplicity-adjusted *p* values. The limited sample size reduces precision and statistical power for modest effects and constrains evaluation of model assumptions. False-discovery-rate control should therefore be interpreted as conditional on the specified model and predefined multiple-testing family.

## 3. Results

### 3.1. Temporal Single-Nucleus Profiling Reveals Early Remodeling of the Renal Cellular Landscape After Ischemic AKI

**Figure 1:**
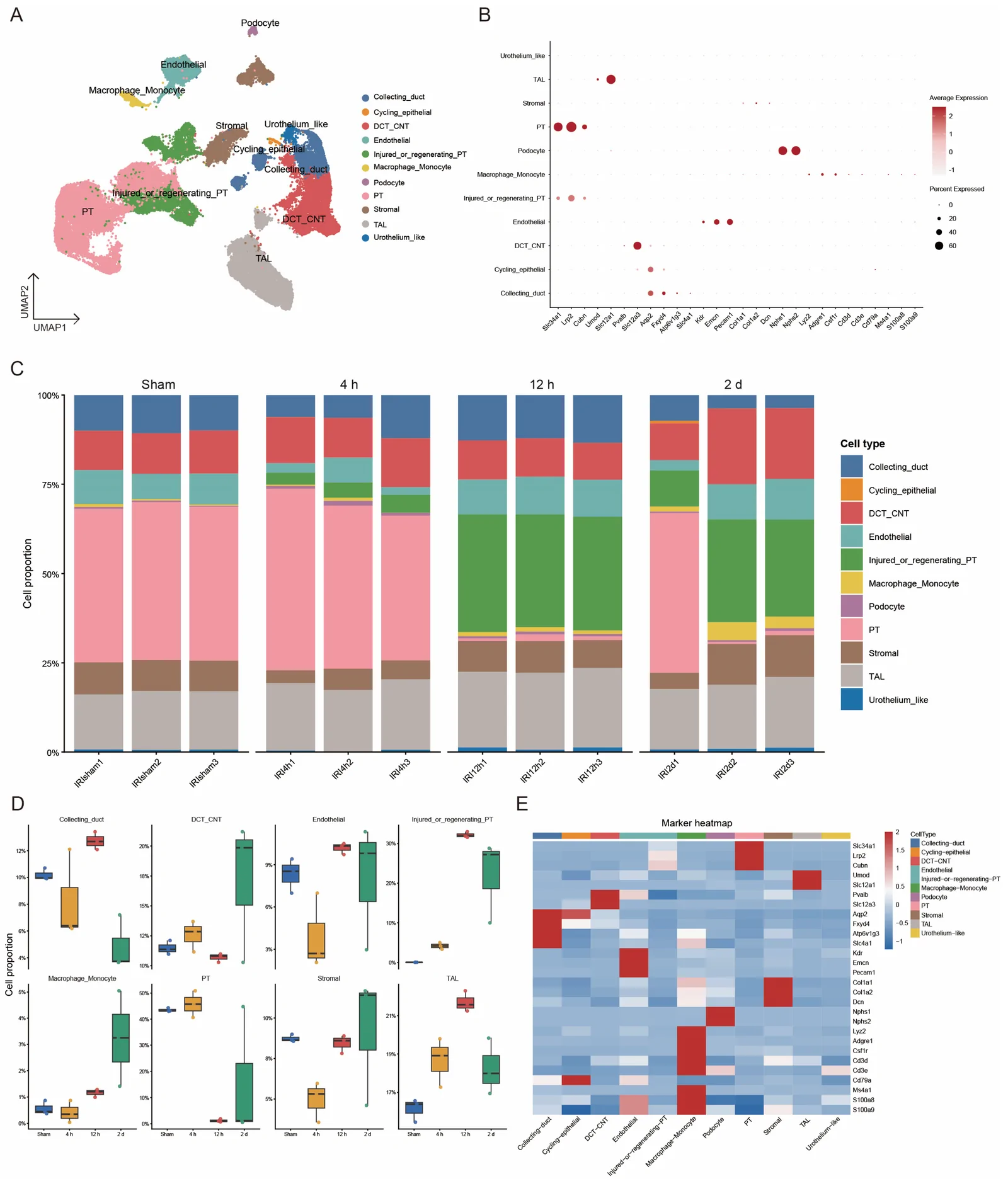
Temporal remodeling of the renal cellular landscape after ischemic acute kidney injury. (**A**) UMAP representation of 79,526 nuclei from 12 independent kidneys showing the 11 annotated renal cell populations. (**B**) Dot plot showing the expression of canonical lineage markers across the annotated populations. (**C**) Sample-level stacked bar plots showing cell-type composition in Sham, 4 h, 12 h, and 2 d kidneys. (**D**) Sample-level proportions of major renal cell populations across time points. (**E**) Heatmap showing representative marker genes for each population. Three independent kidneys were analyzed at each time point.

To characterize the early cellular response to renal ischemia–reperfusion injury, we analyzed 79,526 nuclei from 12 independent kidney samples collected at Sham, 4 h, 12 h, and 2 d, with three biological kidneys included at each time point. Unsupervised clustering and UMAP visualization resolved 11 major renal cell populations, including mature proximal tubular cells, injury- or repair-associated proximal tubular cells, collecting duct cells, distal convoluted and connecting tubular cells, thick ascending limb cells, cycling epithelial cells, urothelium-like cells, endothelial cells, stromal cells, macrophage or monocyte cells, and podocytes (Figure 1A). The annotated populations were supported by established lineage markers. Mature proximal tubular cells expressed *Slc34a1*, *Lrp2*, *Cubn*, and *Slc5a2*. Thick ascending limb cells were enriched for *Umod* and *Slc12a1*, whereas collecting duct cells expressed *Aqp2* and *Fxyd4*. Endothelial cells expressed *Pecam1*, *Emcn*, and *Kdr*; stromal cells expressed *Col1a1*, *Col1a2*, and *Dcn*; macrophage or monocyte cells expressed *Lyz2*, *Adgre1*, and *Csf1r*; and podocytes expressed *Nphs1* and *Nphs2* (Figure 1B). These marker profiles supported the separation of the major epithelial, vascular, stromal, and immune compartments.

Sample-level cell composition was relatively consistent among the three Sham kidneys, in which mature proximal tubular cells represented the dominant epithelial population (Figure 1C and Figure S1). At 4 h, the overall renal cellular architecture remained largely preserved, although early changes were evident in proximal tubular, collecting duct, distal tubular, and endothelial compartments. A more pronounced compositional shift emerged at 12 h, characterized by expansion of the injury- or repair-associated proximal tubular compartment and a corresponding reduction in the mature proximal tubular fraction. Changes were also observed in collecting duct, distal tubular, endothelial, and stromal populations. At 2 d, injury- or repair-associated proximal tubular cells remained abundant, while the proportions of mature proximal tubular, stromal, endothelial, macrophage or monocyte, and other epithelial populations varied among individual kidneys (Figure 1C,D). This inter-sample variability was consistent with heterogeneous entry into the early repair phase. The lineage-marker heatmap further confirmed the distinct transcriptional identities of the annotated renal populations (Figure 1E). Collectively, these findings indicate that ischemic AKI induces rapid remodeling of the renal cellular landscape, with the most prominent early changes occurring within the proximal tubular lineage.

### 3.2. Proximal Tubular Subclustering Identifies Mature, Stressed, Injury-Associated, Repair-Associated, and Cycling States

**Figure 2:**
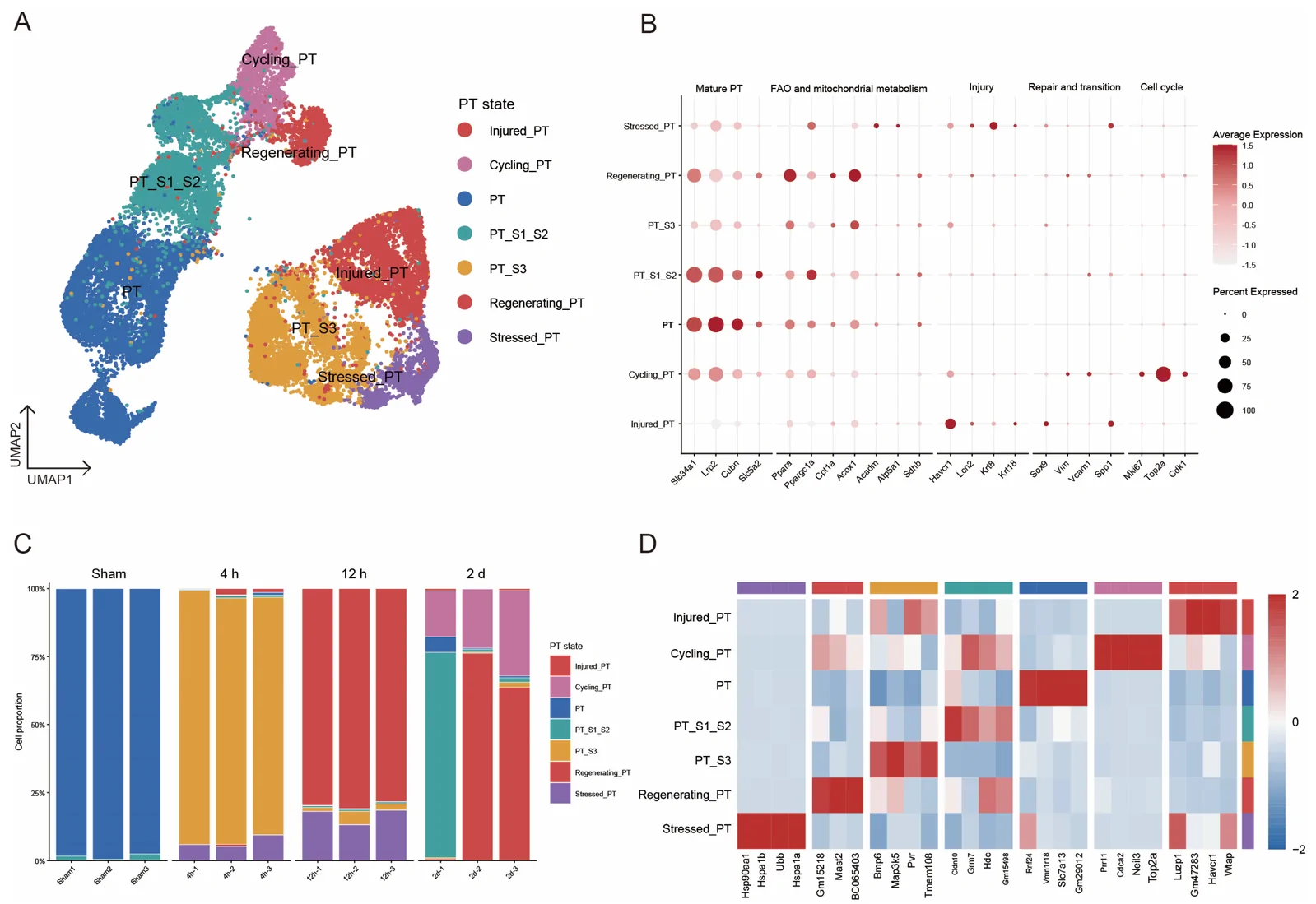
Proximal tubular subclustering identifies distinct segmental and injury-related cell states. (**A**) UMAP representation of 34,151 proximal tubular nuclei showing mature PT, PT_S1_S2, PT_S3, stressed PT, injury-associated PT, repair-associated PT, and cycling PT states. (**B**) Expression of representative segmental, metabolic, injury, repair, and cell-cycle markers. (**C**) Sample-level composition of proximal tubular states across the four time points. (**D**) Heatmap of representative marker genes distinguishing the seven PT states. Three independent kidneys were analyzed at each time point.

Because the whole-kidney analysis identified substantial remodeling of the proximal tubular compartment, we extracted 34,151 proximal tubular nuclei for secondary analysis. UMAP visualization resolved seven transcriptionally distinct PT states: mature PT, PT_S1_S2, PT_S3, stressed PT, injury-associated PT, repair-associated PT, and cycling PT (Figure 2A). Mature PT and PT_S1_S2 cells occupied adjacent regions of the UMAP and retained high expression of differentiated proximal tubular markers, including *Slc34a1*, *Lrp2*, *Cubn*, and *Slc5a2* (Figure 2B). PT_S3 cells displayed a distinct segment-associated transcriptional profile and retained expression of several metabolic genes. Genes associated with fatty acid oxidation and mitochondrial function, including *Ppara*, *Ppargc1a*, *Cpt1a*, *Acox1*, *Acadl*, *Acadm*, *Atp5a1*, and *Sdhb*, were preferentially expressed in mature or metabolically active PT states and were reduced in the most severely injured compartment.

Injury-associated PT cells showed increased expression of *Havcr1*, *Lcn2*, *Krt8*, *Krt18*, *Vcam1*, and *Spp1*. Repair-associated PT cells retained partial proximal tubular identity while expressing epithelial activation, dedifferentiation, and repair-related genes, including *Sox9*, *Vim*, and *Vcam1*. Cycling PT cells were distinguished by strong expression of *Mki67*, *Top2a*, *Cdk1*, and other cell-cycle genes. Stressed PT cells expressed stress-response genes without the full injury-marker profile observed in injury-associated PT cells. These transcriptional patterns indicated that the proximal tubular response to ischemic injury comprised multiple biologically distinct states. The relative abundance of these states changed substantially across the early injury course (Figure 2C). Sham kidneys were composed predominantly of mature PT cells. At 4 h, the proximal tubular compartment shifted toward the PT_S3 state, accompanied by a smaller stressed PT population, consistent with an early segment-associated stress response. At 12 h, repair-associated PT cells became the predominant annotated state, while stressed PT cells remained detectable and smaller fractions of injury-associated and other transitional states persisted. At 2 d, PT-state composition became more heterogeneous among biological samples (Figure S2). One kidney showed expansion of PT_S1_S2 and cycling PT populations, whereas the remaining kidneys retained large repair-associated PT compartments with variable contributions from stressed, injury-associated, and cycling states. The marker heatmap confirmed the transcriptional separation of the seven PT states and highlighted distinct combinations of transport, metabolic, stress, injury, repair, and cell-cycle programs (Figure 2D). These findings define a temporally dynamic proximal tubular state landscape during early ischemic AKI.

### 3.3. Metabolic and Transport Programs Show Maximal Suppression at 12 h After Ischemic Injury

**Figure 3:**
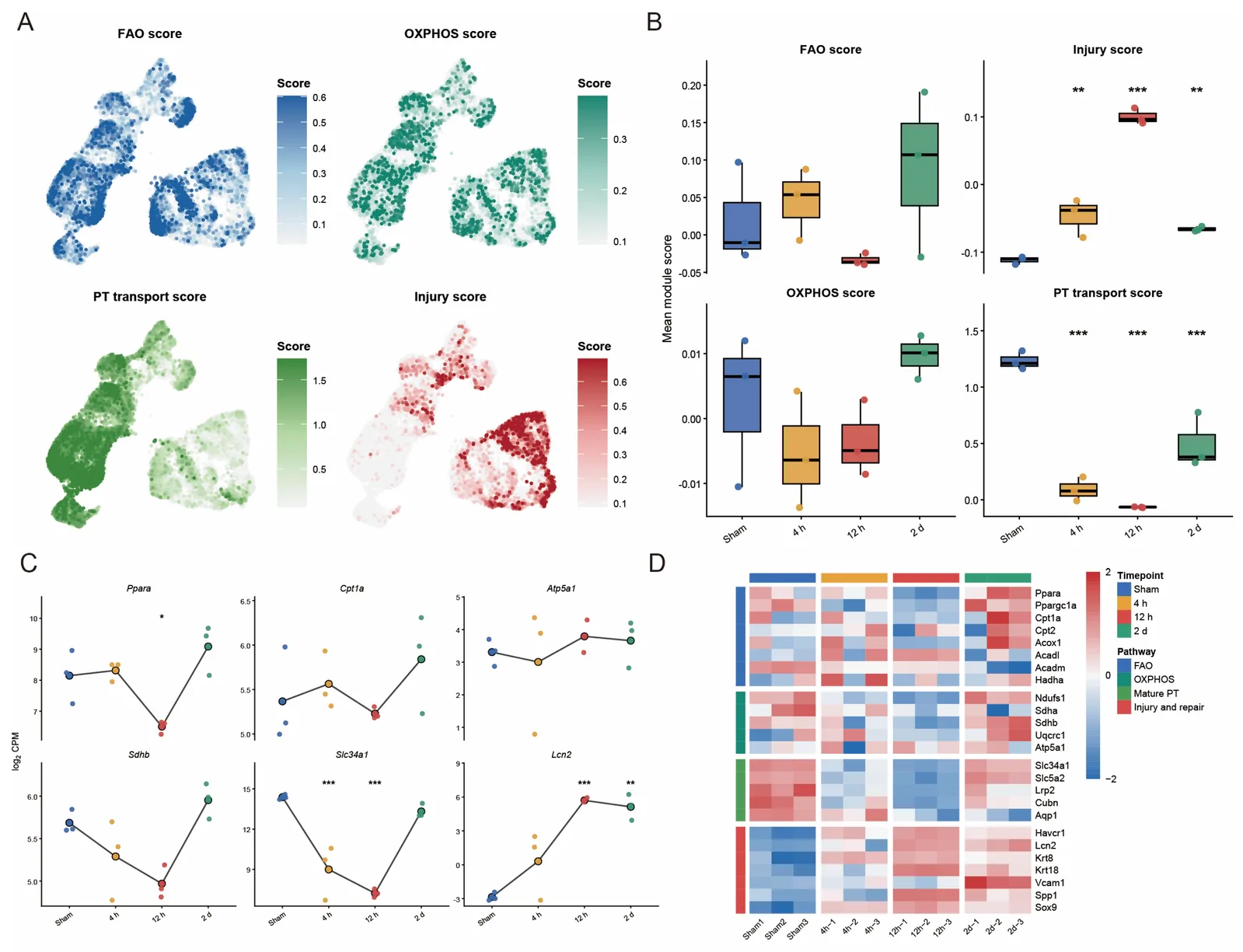
Metabolic, mitochondrial, transport, and injury programs undergo coordinated temporal remodeling. (**A**) UMAP feature plots showing fatty acid oxidation, oxidative phosphorylation, proximal tubular transport, and injury scores. (**B**) Sample-level comparisons of the four functional scores across Sham, 4 h, 12 h, and 2 d. (**C**) Sample-level pseudobulk expression of representative metabolic, mitochondrial, transport, and injury genes. (**D**) Heatmap of pseudobulk expression profiles across the 12 biological samples. Values in the heatmap represent gene-wise standardized expression. Each point represents one independent kidney. Formal effect estimates, confidence intervals, direct adjacent-time contrasts, and sensitivity analyses are provided in Figures S3–S6 and Supplementary Data S2–S6. * *p* < 0.05, ** *p* < 0.01, *** *p* < 0.001.

We next examined whether the observed PT-state remodeling was accompanied by coordinated changes in metabolic competence, mitochondrial function, transport identity, and injury signaling. Fatty acid oxidation, oxidative phosphorylation, proximal tubular transport, and tubular injury scores were projected onto the proximal tubular UMAP (Figure 3A). Fatty acid oxidation and transport scores were highest in mature PT and PT_S1_S2 cells and were lower in stressed, injury-associated, and repair-associated compartments. Oxidative phosphorylation activity showed a broader distribution but was reduced in regions enriched for stressed and injury-associated PT cells. In contrast, the injury score was concentrated in the injury-associated PT compartment and was also elevated in subsets of stressed and repair-associated cells. Sample-level comparisons, based on three independent kidneys at each time point, revealed distinct temporal patterns in these functional programs (Figure 3B). The fatty acid oxidation score showed a modest increase at 4 h, reached its lowest level at 12 h, and showed partial restoration at 2 d. Oxidative phosphorylation was reduced at 4 h and remained low at 12 h, followed by a partial rebound at 2 d. The proximal tubular transport score decreased at both 4 h and 12 h and showed only partial restoration at 2 d. In contrast, the injury score increased at 4 h, reached its highest level at 12 h, and declined at 2 d, although it remained above the Sham level. The cell-level UMAP projections and score distributions across annotated PT states are presented as descriptive summaries and are not interpreted as independent validation of the state annotations; formal temporal inference is based on biological-kidney-level analyses.

Sample-level pseudobulk analysis supported these functional patterns (Figure 3C). *Ppara* and *Cpt1a* expression was relatively preserved at 4 h, decreased at 12 h, and moved toward the Sham level at 2 d. *Sdhb* showed a similar reduction during peak injury, whereas *Slc34a1* was strongly suppressed at 4 h and 12 h and showed partial re-expression at 2 d. In contrast, *Lcn2* was prominently induced at 12 h and remained elevated at 2 d. *Atp5a1* transcript abundance showed comparatively modest temporal variation. A broader pseudobulk heatmap demonstrated coordinated temporal regulation of fatty acid oxidation, oxidative phosphorylation, mature proximal tubular identity, and injury- or repair-associated gene sets (Figure 3D). Fatty acid oxidation genes, including *Ppara*, *Ppargc1a*, *Cpt1a*, *Cpt2*, *Acox1*, *Acadl*, *Acadm*, and *Hadha*, were generally reduced at peak injury and partially restored in the 2 d samples. Mitochondrial genes, including *Ndufs1*, *Sdha*, *Sdhb*, *Uqcrc1*, and *Atp5a1*, displayed a related pattern of early disruption and partial later restoration. Mature PT markers, including *Slc34a1*, *Slc5a2*, *Lrp2*, *Cubn*, and *Aqp1*, were suppressed during acute injury, whereas *Havcr1*, *Lcn2*, *Krt8*, *Krt18*, *Vcam1*, *Spp1*, and *Sox9* were induced. Together, these results show that early PT-state remodeling is temporally associated with loss of fatty acid oxidation, mitochondrial, and transport programs and with induction of injury-related transcriptional activity. The strongest combined metabolic and functional disruption was observed at 12 h. To formally quantify these temporal changes, we additionally examined effect estimates, confidence intervals, direct adjacent-time contrasts, and pathway-level sensitivity analyses (Figures S3–S6 and Supplementary Data S2–S6). Importantly, the principal pathway findings were also examined after exclusion of annotation/review genes, and the key FAO- and transport-related temporal patterns remained directionally consistent in these sensitivity analyses. Ppara was reduced at 12 h relative to Sham and increased at 2 d relative to 12 h (Figure S4), whereas Cpt1a did not reach the prespecified gene-level FDR threshold in any of the five primary contrasts. Fatty acid metabolism and mitochondrial FAO gene sets were negatively enriched at 12 h relative to 4 h, whereas Hallmark oxidative phosphorylation did not show significant negative enrichment. SLC-mediated transport showed suppression during acute injury and partial recovery at 2 d (Figure S6). These results indicate program-specific temporal remodeling rather than uniform suppression of all metabolic pathways.

### 3.4. Trajectory Analyses Associate Loss of Metabolic Competence with Proximal Tubular State Remodeling

**Figure 4:**
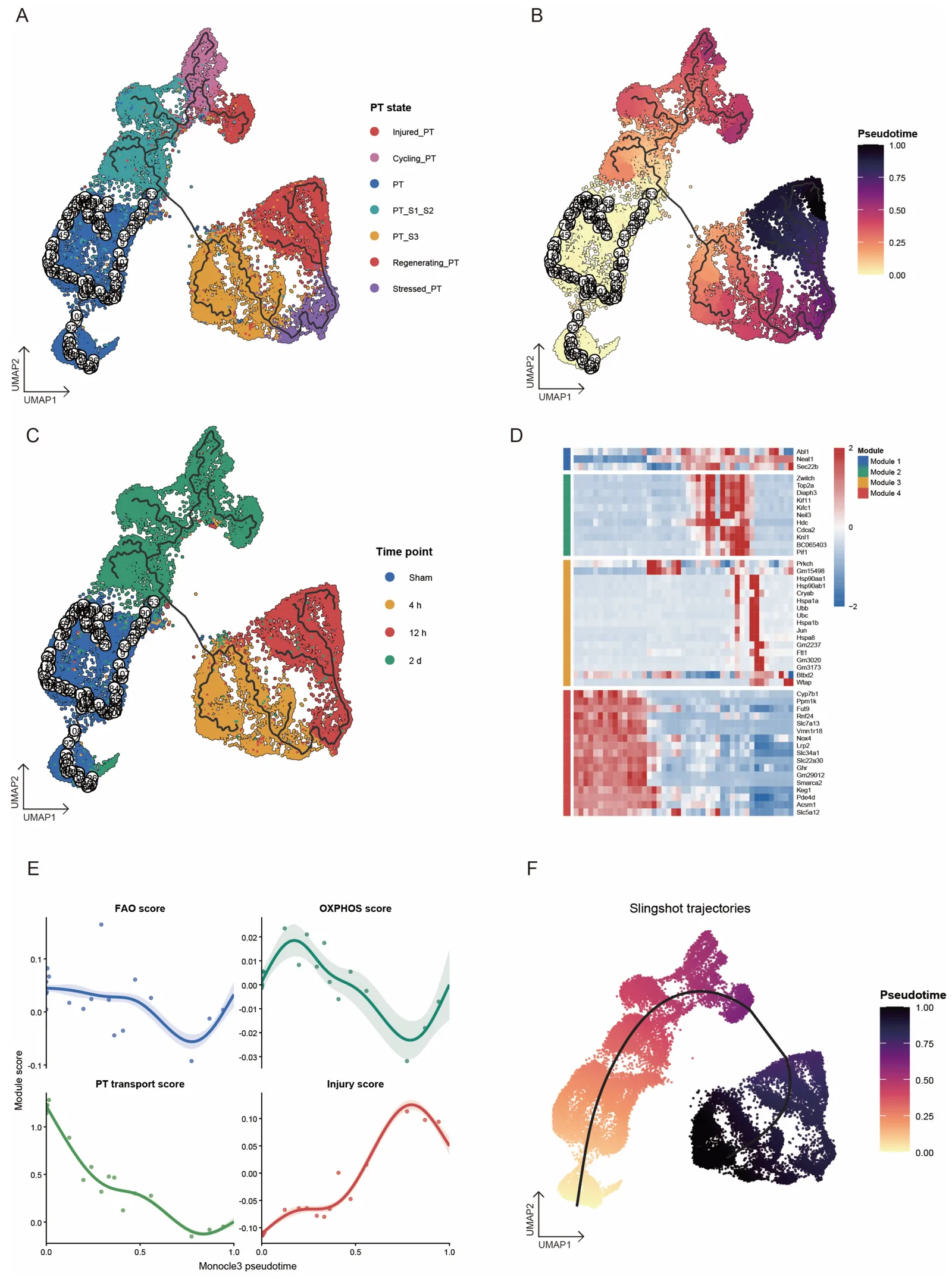
Trajectory analyses associate metabolic decline with proximal tubular state remodeling. (**A**) Monocle3 principal graph overlaid on the proximal tubular UMAP and colored by PT state. (**B**) Monocle3 pseudotime distribution. (**C**) Distribution of experimental time points along the inferred trajectory. (**D**) Heatmap of the top pseudotimeassociated genes grouped into four dynamic expression modules. (**E**) Sample-aware generalized additive model fits showing changes in fatty acid oxidation, oxidative phosphorylation, transport, and injury scores along pseudotime. Shaded regions represent 95% confidence intervals. (**F**) Slingshot lineage curves showing a complementary trajectory ordering. Monocle3 and Slingshot pseudotime values were positively correlated at the cell level. Additional trajectory-coverage and root-sensitivity analyses are provided in Figure S7 and Supplementary Data S7.

To examine transcriptional relationships among the identified PT states, we performed trajectory inference using Monocle3. The principal graph was rooted in the mature PT compartment, which was enriched in Sham kidneys and expressed high levels of differentiated proximal tubular markers. The inferred trajectory extended from mature PT and segment-specific states toward stressed, repair-associated, cycling, and injury-associated compartments (Figure 4A). Pseudotime values were lowest in mature PT cells and increased across transitional and stress-related regions of the UMAP (Figure 4B). Repair-associated and cycling PT cells were located predominantly at intermediate pseudotime positions, whereas injury-associated PT cells were enriched at later positions. This computational ordering was consistent with a transition from differentiated proximal tubular identity toward stress-, repair-, and injury-associated transcriptional states. The distribution of experimental time points along the trajectory broadly corresponded to the biological injury course (Figure 4C). Sham nuclei were concentrated near the trajectory origin. Nuclei collected at 4 h were enriched in early transitional regions containing PT_S3 and stressed PT cells. Nuclei collected at 12 h were concentrated in repair-associated and injury-associated regions, whereas 2 d nuclei were distributed across later repair-associated, cycling, and partially restored compartments. The overlap among time points indicated that elapsed experimental time and the transcriptional state were related but not equivalent.

Dynamic-gene analysis identified four major expression modules along pseudotime (Figure 4D). Genes associated with differentiated proximal tubular transport and metabolic function were enriched at early pseudotime and declined across the inferred trajectory. Other modules showed intermediate or late induction and contained stress-response, cell-cycle, injury, and repair-associated genes. Cell-cycle genes, including *Top2a* and related mitotic regulators, were induced within a restricted intermediate-to-late interval, whereas mature PT genes such as *Lrp2* and *Slc34a1* declined toward later pseudotime positions. Generalized additive modeling further demonstrated distinct pseudotime-associated patterns in functional programs (Figure 4E). Fatty acid oxidation remained relatively preserved at early pseudotime and declined across intermediate and later positions, with partial recovery at the latest positions. Oxidative phosphorylation showed a biphasic pattern, with early preservation or modest elevation followed by decline and partial late restoration. The proximal tubular transport score decreased progressively across pseudotime, whereas the injury score increased during the second half of the trajectory and was highest in later injury-associated regions. Slingshot was used as a complementary trajectory-inference approach and produced a similar ordering from mature PT through transitional and repair-associated states toward injury-associated compartments (Figure 4F). Monocle3 and Slingshot pseudotime values were positively correlated at the cell level (Spearman ρ = 0.73, two-sided *p* < 0.001), and the median ordering of the annotated PT states was concordant between the two methods. These analyses support a reproducible association between loss of differentiated metabolic and transport programs and the emergence of stress-, repair-, and injury-associated PT states (Figure S7).

### 3.5. Exploratory CellChat Analysis Reveals Time-Dependent Remodeling of Predicted Renal Intercellular Communication During Early AKI

**Figure 5:**
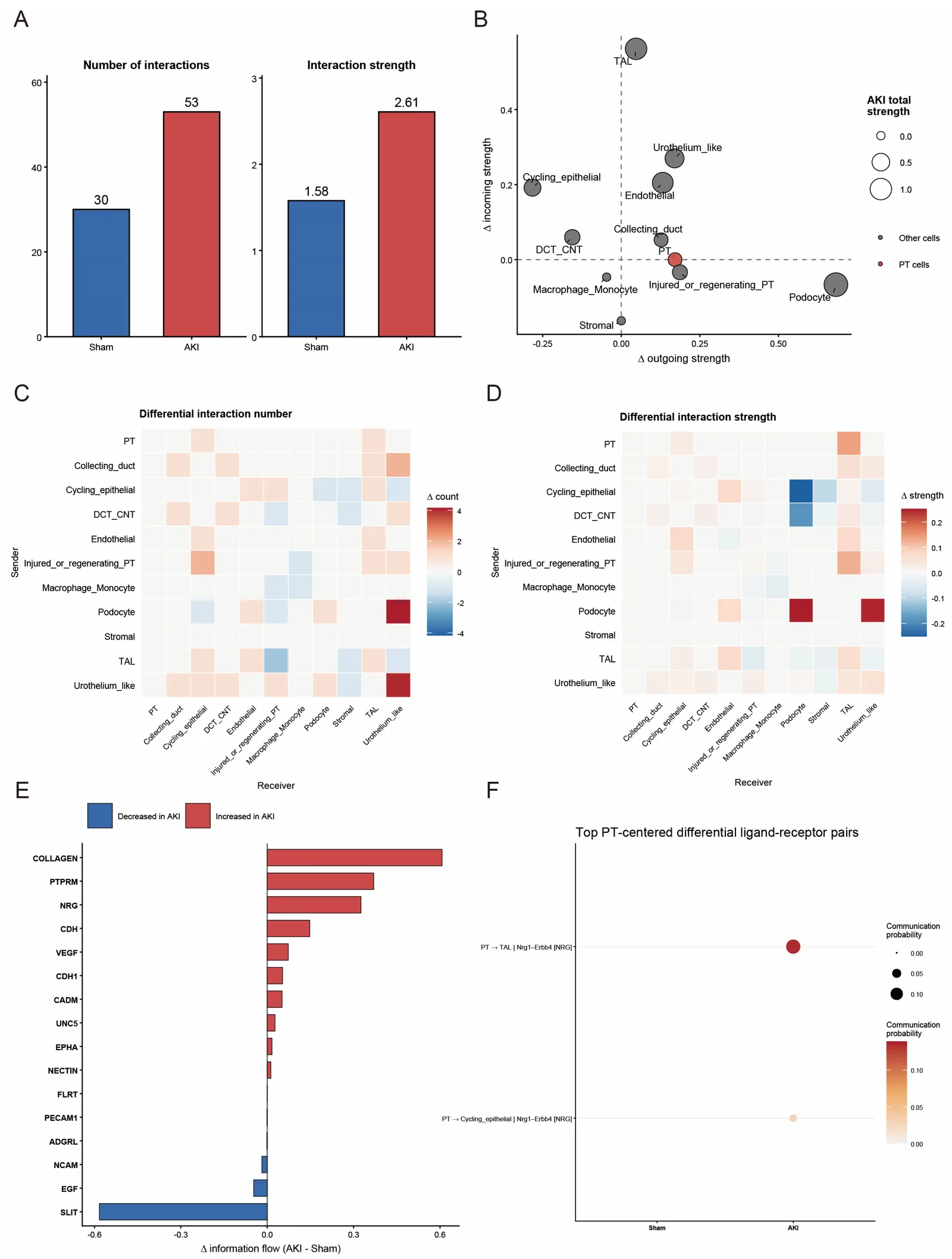
Exploratory CellChat analysis predicts remodeling of renal intercellular communication during early acute kidney injury. (**A**) Total number and aggregate strength of predicted interactions in Sham and pooled early-AKI networks. (**B**) Changes in outgoing and incoming signaling strength across renal cell populations. (**C**) Differential sender–receiver interaction-number matrix. (**D**) Differential interaction-strength matrix. (**E**) Differences in pathway-level information flow between pooled early AKI and Sham. (**F**) Selected PT-centered ligand-receptor interactions, including predicted Nrg1–Erbb4 signaling. Positive values indicate greater predicted communication in early AKI. These pooled analyses are retained as a descriptive overview of the early-AKI communication landscape. Time-specific CellChat analyses for Sham, 4 h, 12 h, and 2 d, together with individual-kidney support assessments, are provided in Supplementary Figure S8 and Supplementary Data S8–S11.

To explore whether proximal tubular remodeling occurred within a broader alteration of the renal signaling environment, we compared CellChat-inferred communication networks between Sham kidneys and pooled early-AKI samples. Because the 4 h, 12 h, and 2 d samples were combined for this analysis, the resulting networks were interpreted as an integrated and exploratory representation of early AKI. The pooled early-AKI network contained 53 predicted interactions compared with 30 in the Sham network, while aggregate communication strength increased from 1.58 to 2.61 (Figure 5A). Analysis of outgoing and incoming signaling roles indicated cell-type-specific network remodeling (Figure 5B). Thick ascending limb cells showed a pronounced increase in incoming signaling with comparatively little change in outgoing strength. Urothelium-like and endothelial populations showed increases in both outgoing and incoming signaling, whereas cycling epithelial cells showed lower outgoing and higher incoming signaling. Mature PT and injury- or repair-associated PT compartments occupied distinct positions within the early-AKI communication network. Differential interaction-number and interaction-strength matrices indicated that the predicted changes were concentrated within selected sender–receiver relationships (Figure 5C,D). Prominent alterations involved urothelium-like cells, podocytes, thick ascending limb cells, collecting duct cells, injury- or repair-associated PT cells, and cycling epithelial populations. Some sender–receiver pairs showed reduced interaction number or strength, indicating redistribution of the predicted communication network rather than a uniform increase across all cell types.

Pathway-level analysis predicted increased information flow through collagen, PTPRM, NRG, cadherin, VEGF, CDH1, CADM, UNC5, EPHA, NECTIN, FLRT, PECAM1, and ADGRL signaling in early AKI, whereas NCAM, EGF, and SLIT signaling showed lower information flow (Figure 5E). Collagen signaling exhibited the largest positive difference, consistent with early extracellular-matrix and epithelial-stromal remodeling. Alterations in cadherin, CADM, NECTIN, PTPRM, and PECAM1 signaling further suggested changes in cell-adhesion and tissue-organization programs. Among PT-centered candidate interactions, Nrg1-Erbb4 signaling from PT cells to thick ascending limb and cycling epithelial populations was selectively detected or strengthened in the pooled early-AKI network (Figure 5F). The predicted PT-to-thick ascending limb interaction was stronger than the corresponding PT-to-cycling epithelial interaction. Nrg1-Erbb4 therefore emerged as a candidate component of altered cross-nephron epithelial signaling. Because these results were inferred from transcript expression in pooled samples, they require spatial, protein-level, and functional validation. To further resolve temporal communication dynamics, we additionally performed CellChat analyses separately for Sham, 4 h, 12 h, and 2 d, while maintaining a common set of analyzable cell populations and sender–receiver relationships across time points. The time-specific networks contained 121, 203, 321, and 209 predicted ligand-receptor relationships at Sham, 4 h, 12 h, and 2 d, respectively. Of these, 89, 96, 252, and 153 relationships, respectively, were also detected in at least two evaluable kidneys. These network totals are descriptive and were not subjected to biological-kidney between-condition significance testing. The 12 h network showed the largest number of predicted relationships within the common comparison framework, whereas the 2 d network displayed a distinct pattern rather than simply retaining the 12 h communication profile. Time-specific analysis also refined the interpretation of PT-centered candidate interactions. The predicted Nrg1/Erbb4 relationship from PT cells to thick ascending limb populations was detected from 4 h onward and persisted through 12 h and 2 d, indicating that this candidate relationship was not specific to the 12 h injury stage. PT-to-CTAL1, PT-to-CTAL2, and PT-to-MTAL Nrg1/Erbb4 relationships were repeatedly detected across evaluable injured kidneys, whereas they were not detected in the corresponding Sham kidney models. In parallel, PT pseudobulk analysis showed increased Nrg1 expression at the injury time points relative to Sham, while Erbb4 expression was already broadly detectable in the recipient thick ascending limb populations under Sham conditions. These observations therefore support a time-associated change in predicted PT-to-thick ascending limb communication without implying simultaneous ligand and receptor induction or experimentally validated pathway activation. The complete time-specific comparisons, individual-kidney support summaries, and Nrg1/Erbb4 expression analyses are provided in Supplementary Figure S8 and Supplementary Data S8–S11.

### 3.6. Ischemic AKI Suppresses PPARα/CPT1A-Associated Metabolic Proteins and Induces Tubular Injury In Vivo

**Figure 6:**
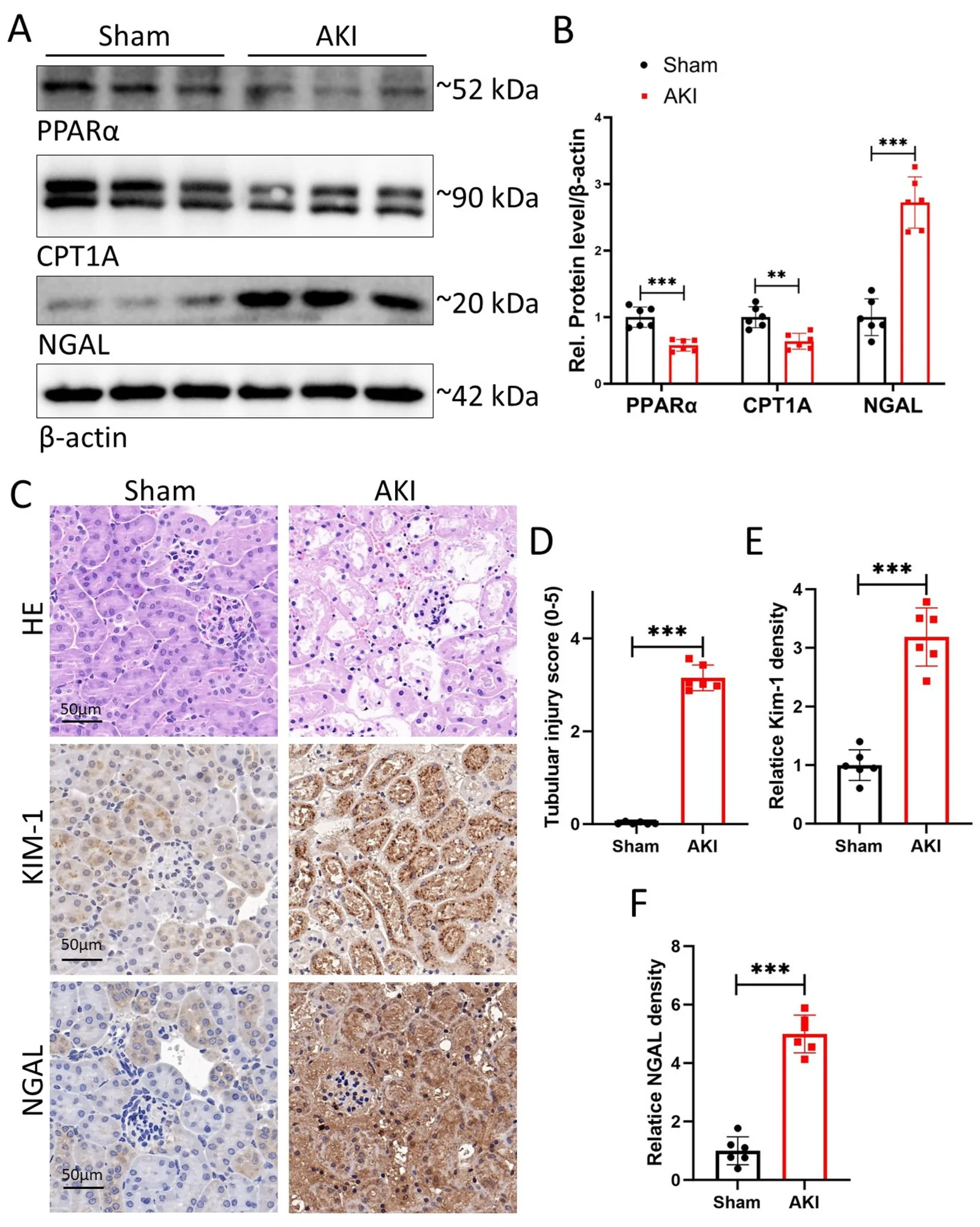
Ischemic acute kidney injury suppresses PPARα and CPT1A expression and induces tubular injury in vivo. (**A**) Representative Western blots showing PPARα, CPT1A, NGAL, and β-actin expression in kidney tissues collected at 24 h after reperfusion from male Sham and AKI mice. (**B**) Densitometric quantification of PPARα, CPT1A, and NGAL protein expression normalized to β-actin. (**C**) Representative hematoxylin and eosin staining and immunohistochemical staining for KIM-1 and NGAL in kidney sections. (**D**) Semiquantitative tubular injury scores. (**E**) Quantification of KIM-1 staining as relative mean optical density. (**F**) Quantification of NGAL staining as relative mean optical density. Data are presented as mean ± SD; *n* = 6 male mice per group. Each data point represents one independent animal. Each data point represents one independent mouse. Statistical comparisons were performed using a two-tailed unpaired Student’s *t* test. Exact *p* values were as follows: PPARα, *p* = 0.0006; CPT1A, *p* = 0.0042; NGAL protein, *p* = 0.0003; tubular injury score, *p* = 0.0002; KIM-1 staining, *p* = 0.0004; and NGAL staining, *p* = 0.0007. ** *p* < 0.01; *** *p* < 0.001. Scale bars, 50 μm.

We next assessed selected transcriptomic observations at a separate 24-h reperfusion endpoint in a male C57BL/6J mouse model of renal ischemia–reperfusion injury. Western blotting showed that renal PPARα and CPT1A protein abundance was lower in AKI kidneys than in Sham kidneys, whereas NGAL abundance was increased (Figure 6A,B). Densitometric analysis across six independent animals per group confirmed reductions in PPARα and CPT1A and an increase in NGAL. PPARα was reduced at *p* < 0.001, CPT1A at *p* < 0.01, and NGAL was increased at *p* < 0.001. Histological examination showed preserved tubular architecture and brush-border morphology in Sham kidneys. AKI kidneys displayed tubular dilation, epithelial flattening, cytoplasmic vacuolization, brush-border loss, luminal debris, and increased interstitial cellularity (Figure 6C). Semiquantitative analysis confirmed a higher tubular injury score in AKI mice than in Sham controls (*p* < 0.001; Figure 6D). KIM-1 immunostaining was weak in Sham kidneys and strongly induced in damaged tubular regions after ischemia–reperfusion injury. Quantitative analysis demonstrated an increase in KIM-1 staining density in AKI kidneys (*p* < 0.001; Figure 6E). NGAL immunostaining was similarly low in Sham kidneys and increased throughout injured tubular regions after AKI, with quantitative analysis confirming higher NGAL staining density (*p* < 0.001; Figure 6F). These findings provide cross-sectional protein- and tissue-level support for selected injury-associated molecular changes at 24 h; they do not directly validate the temporal kinetics identified across the 4 h, 12 h, and 2 d transcriptomic samples.

### 3.7. AKI Reduces Mitochondrial Proteins and Disrupts Differentiated Proximal Tubular Identity

**Figure 7:**
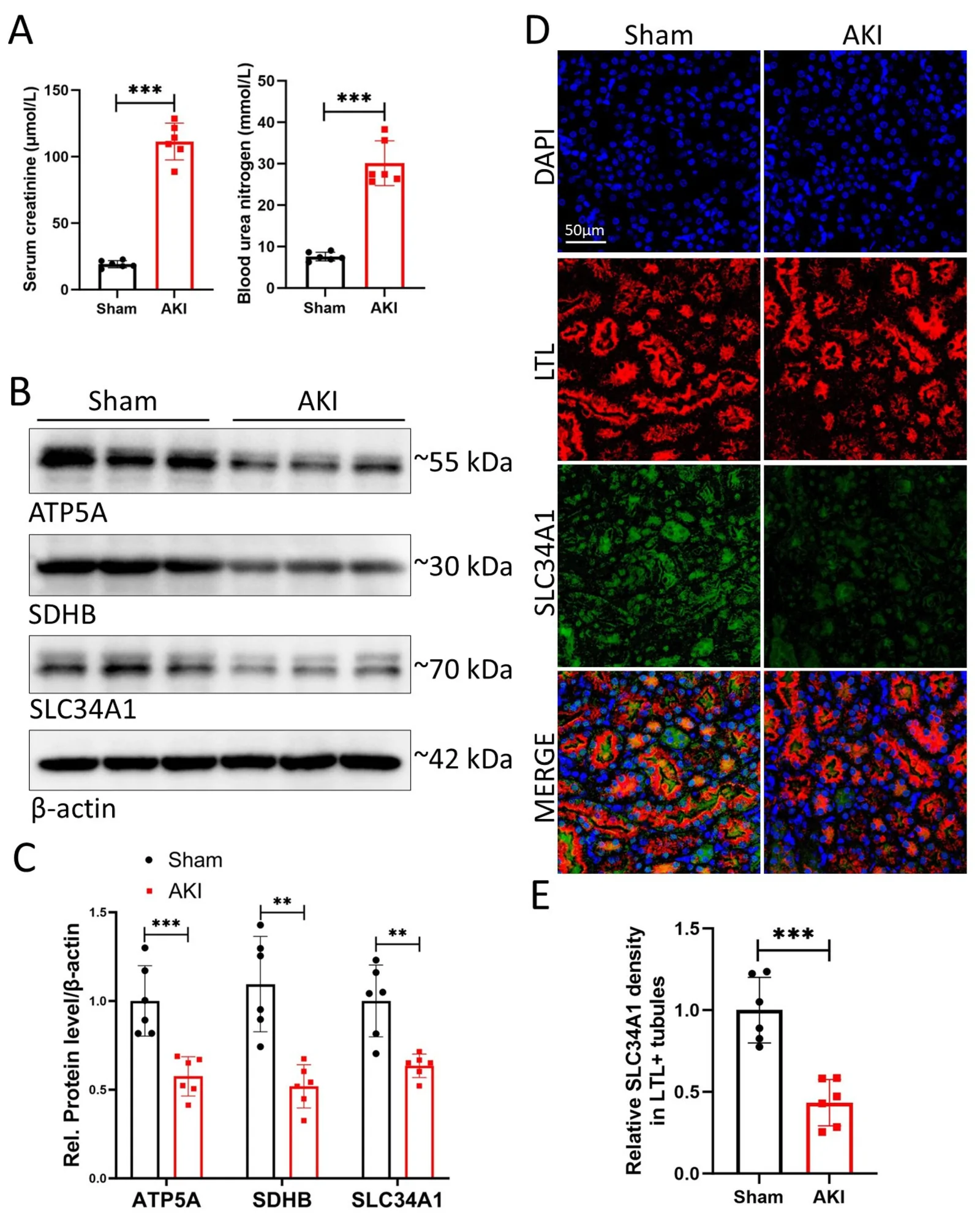
Acute kidney injury impairs renal function, reduces mitochondrial proteins, and disrupts differentiated proximal tubular identity. Kidney tissues and serum were collected from male mice at 24 h after reperfusion. (**A**) Serum creatinine and blood urea nitrogen levels in Sham and AKI mice. (**B**) Representative Western blots showing ATP5A, SDHB, SLC34A1, and β-actin expression in kidney tissues. Three representative animals from each group are shown. (**C**) Densitometric quantification of ATP5A, SDHB, and SLC34A1 protein expression normalized to β-actin. (**D**) Representative immunofluorescence images showing DAPI, LTL, SLC34A1, and merged signals in kidney sections from Sham and AKI mice. (**E**) Quantification of SLC34A1 mean fluorescence intensity within LTL-positive proximal tubular regions. Data are presented as mean ± SD; *n* = 6 male mice per group. Each data point represents one independent animal. Each data point represents one independent mouse. Statistical comparisons were performed using a two-tailed unpaired Student’s *t* test. Exact *p* values were as follows: serum creatinine, *p* = 0.0003; blood urea nitrogen, *p* = 0.0005; ATP5A, *p* = 0.0008; SDHB, *p* = 0.0061; SLC34A1 protein, *p* = 0.0048; and SLC34A1 fluorescence intensity, *p* = 0.0006. ** *p* < 0.01; *** *p* < 0.001. Scale bars, 50 μm.

Renal dysfunction was evaluated by measuring serum creatinine and blood urea nitrogen levels. Both parameters were higher in male AKI mice than in Sham controls (*p* < 0.001 for both; Figure 7A), confirming substantial impairment of renal function after ischemia–reperfusion injury. Western blotting showed lower renal ATP5A, SDHB, and SLC34A1 protein abundance in the AKI group (Figure 7B,C). ATP5A was reduced at *p* < 0.001, whereas SDHB and SLC34A1 were reduced at *p* < 0.01. The simultaneous reduction in ATP5A and SDHB expression was consistent with impaired mitochondrial oxidative metabolism, while the loss of SLC34A1 indicated disruption of differentiated proximal tubular transport identity. Immunofluorescence analysis further evaluated SLC34A1 expression within LTL-positive proximal tubules (Figure 7D). Sham kidneys showed strong SLC34A1 fluorescence within LTL-positive tubular regions and extensive overlap between SLC34A1 and the proximal tubular brush-border marker. In AKI kidneys, LTL-positive structures remained detectable, while SLC34A1 fluorescence was reduced and showed greater spatial heterogeneity. Quantification of SLC34A1 fluorescence within LTL-positive regions confirmed a significant reduction in AKI kidneys (*p* < 0.001; Figure 7E). Together, these findings connect renal functional impairment with loss of mitochondrial proteins and mature proximal tubular transport identity. The tissue-level findings were directionally consistent with selected mitochondrial and transport-related alterations identified in the transcriptomic analysis. However, these measurements were obtained at the separate 24-h endpoint and therefore do not establish the timing of the transcriptomic changes.

### 3.8. Hypoxia–Reoxygenation Reproduces PPARα/CPT1A Suppression and Apoptotic Signaling in Tubular Epithelial Cells

**Figure 8:**
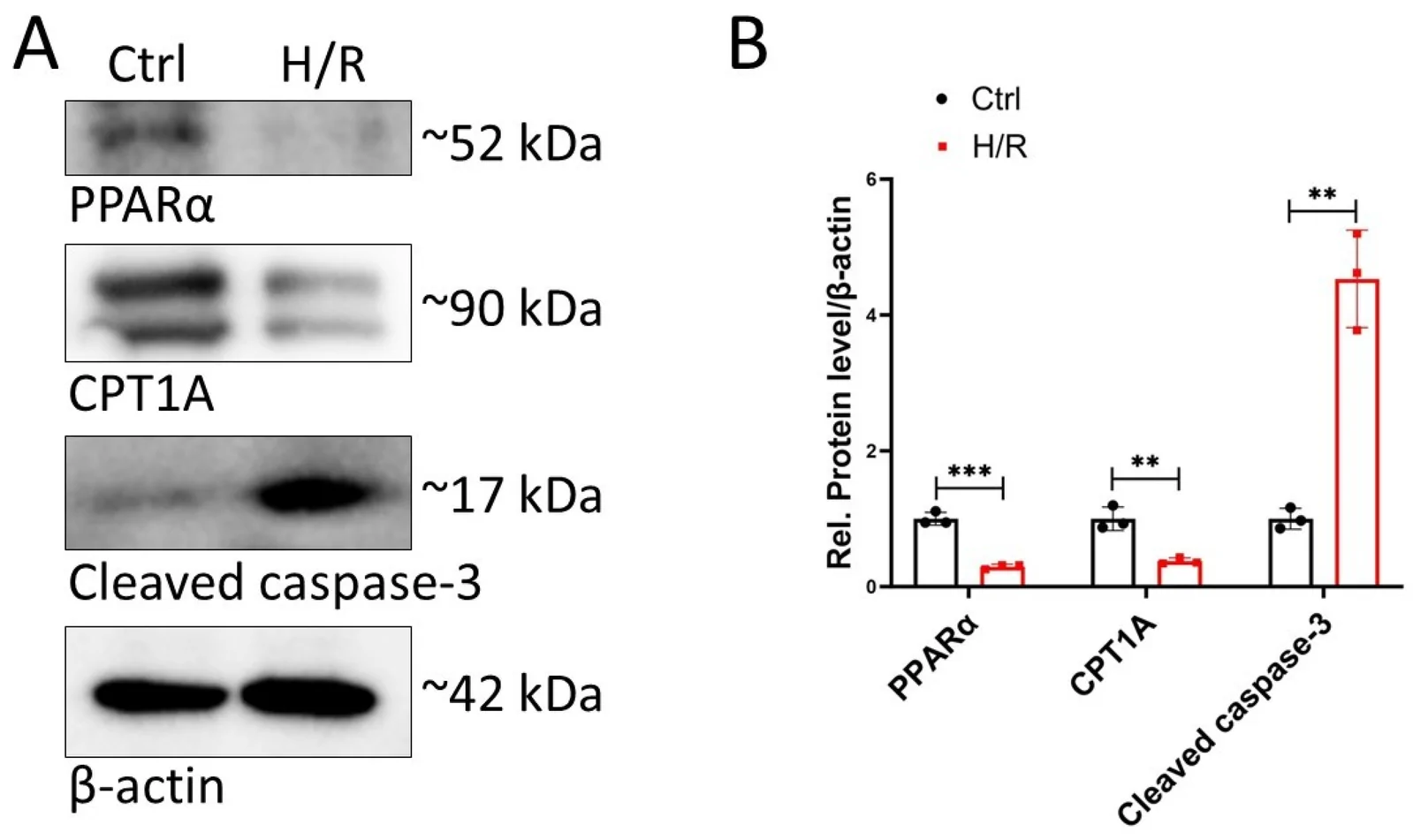
Hypoxia–reoxygenation suppresses PPARα and CPT1A expression and activates apoptotic signaling in tubular epithelial cells. (**A**) Representative Western blots showing PPARα, CPT1A, cleaved caspase-3, and β-actin expression in HK-2 cells maintained under normoxic control conditions or subjected to hypoxia–reoxygenation. (**B**) Densitometric quantification of PPARα, CPT1A, and cleaved caspase-3 protein expression normalized to β-actin. Data are presented as mean ± SD from three independent biological experiments. Each data point represents one independent experiment. Statistical comparisons were performed using a two-tailed unpaired Student’s *t* test. Exact *p* values were *p* = 0.0007 for PPARα, *p* = 0.0054 for CPT1A, and *p* = 0.0039 for cleaved caspase-3. ** *p* < 0.01; *** *p* < 0.001. Ctrl, normoxic control; H/R, hypoxia–reoxygenation.

To determine whether hypoxia–reoxygenation stress could reproduce key features of the in vivo metabolic phenotype, tubular epithelial cells were subjected to hypoxia followed by reoxygenation. Western blotting showed lower PPARα and CPT1A protein abundance and higher cleaved caspase-3 abundance after hypoxia–reoxygenation than under normoxic conditions (Figure 8A). Quantitative analysis of three independent biological experiments confirmed a reduction in PPARα following hypoxia–reoxygenation (*p* < 0.001; Figure 8B). CPT1A was also reduced (*p* < 0.01), while cleaved caspase-3 was increased (*p* < 0.01). These findings indicate that hypoxia–reoxygenation is sufficient to reproduce the association between PPARα/CPT1A suppression and activation of apoptotic signaling observed in ischemic kidney injury. Collectively, the transcriptomic analyses define time-associated changes across the early injury course, whereas the animal and cell-based experiments provide complementary cross-sectional and functional evidence at distinct experimental levels.

### 3.9. Pharmacological Activation of PPARα Attenuates H/R-Induced Mitochondrial Dysfunction and Cellular Injury in a CPT1A-Dependent Manner

**Figure 9:**
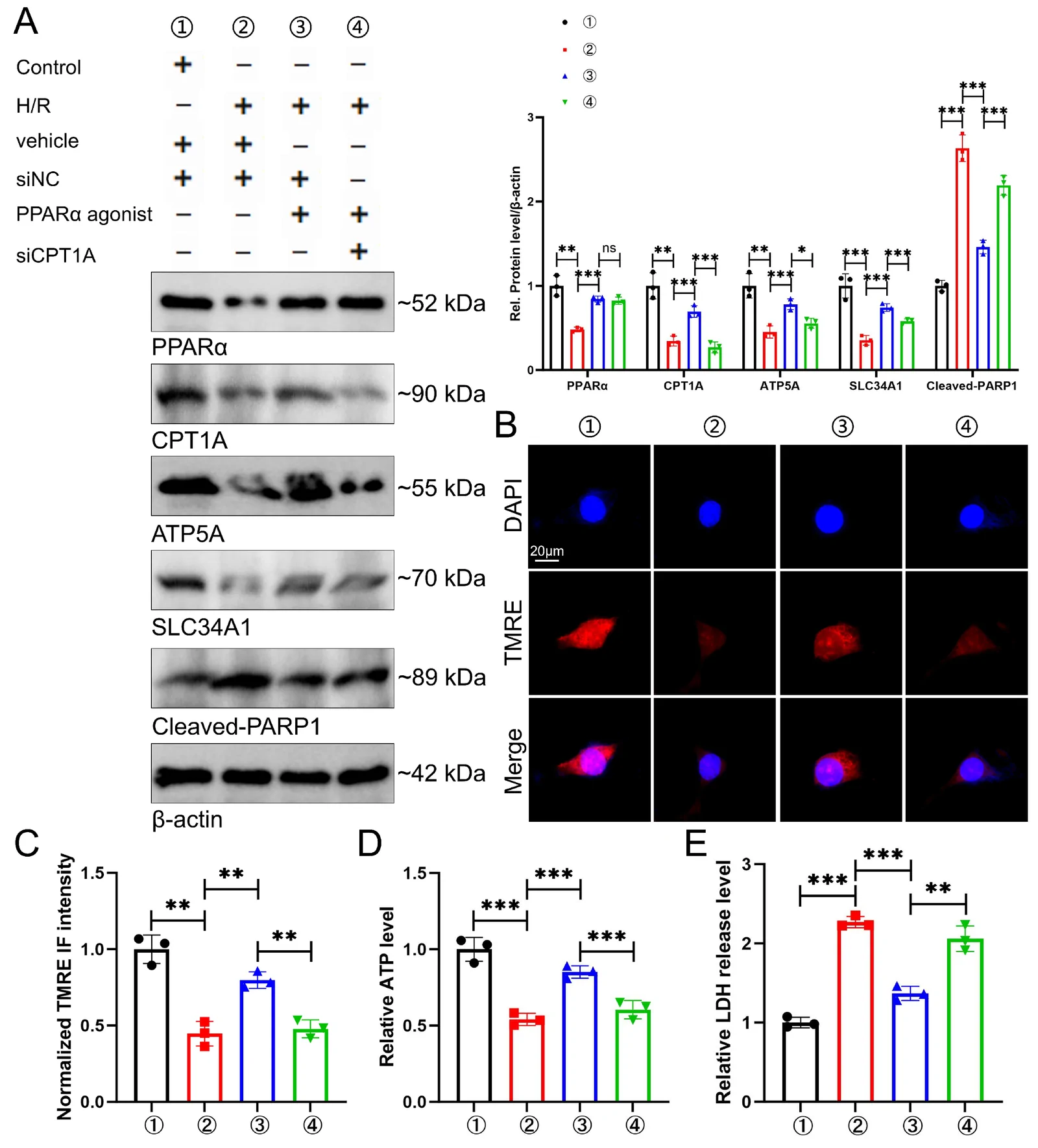
PPARα activation attenuates H/R-induced mitochondrial dysfunction and cellular injury in HK-2 cells partly through CPT1A. HK-2 cells were assigned to four experimental groups: ① control + vehicle + siNC, ② H/R + vehicle + siNC, ③ H/R + PPARα agonist + siNC, and ④ H/R + PPARα agonist + siCPT1A. (**A**) Representative Western blots and densitometric quantification of PPARα, CPT1A, ATP5A, SLC34A1, and cleaved PARP1 protein levels. β-actin was used as the loading control. H/R reduced PPARα, CPT1A, ATP5A, and SLC34A1 expression and increased cleaved PARP1. PPARα agonist treatment partially restored CPT1A, ATP5A, and SLC34A1 expression and reduced cleaved PARP1, whereas CPT1A knockdown attenuated these effects. (**B**) Representative fluorescence images of mitochondrial membrane potential assessed by TMRE staining. Nuclei were counterstained with DAPI. Scale bar, 20 μm. (**C**) Quantification of normalized TMRE fluorescence intensity. H/R reduced mitochondrial membrane potential, which was partially restored by PPARα agonist treatment; this improvement was attenuated by CPT1A knockdown. (**D**) Relative intracellular ATP levels in the four experimental groups. H/R decreased cellular ATP levels, whereas PPARα activation partially restored ATP production; CPT1A knockdown diminished this recovery. (**E**) Relative LDH release. H/R increased LDH release, which was reduced by PPARα agonist treatment and increased again following CPT1A knockdown. Data are presented as mean ± SD from three independent experiments (*n* = 3), with individual data points shown. Statistical comparisons were performed using one-way ANOVA followed by Tukey’s multiple-comparisons test. For the principal comparisons, the exact Tukey-adjusted *p* values were as follows. For H/R + vehicle + siNC versus control + vehicle + siNC: PPARα, *p* = 0.0008; CPT1A, *p* = 0.0026; ATP5A, *p* = 0.0009; SLC34A1, *p* = 0.0017; cleaved PARP1, *p* = 0.0006; TMRE fluorescence, *p* = 0.0004; ATP, *p* = 0.0008; and LDH release, *p* = 0.0003. For H/R + WY-14643 + siNC versus H/R + vehicle + siNC: PPARα, *p* = 0.214; CPT1A, *p* = 0.0041; ATP5A, *p* = 0.0068; SLC34A1, *p* = 0.0085; cleaved PARP1, *p* = 0.0059; TMRE fluorescence, *p* = 0.0037; ATP, *p* = 0.0072; and LDH release, *p* = 0.0029. For H/R + WY-14643 + siCPT1A versus H/R + WY-14643 + siNC: PPARα, *p* = 0.683; CPT1A, *p* = 0.0016; ATP5A, *p* = 0.0124; SLC34A1, *p* = 0.0186; cleaved PARP1, *p* = 0.0152; TMRE fluorescence, *p* = 0.0108; ATP, *p* = 0.0165; and LDH release, *p* = 0.0131. *p* < 0.05, *p* < 0.01, *p* < 0.001; ns, not significant. Ctrl, normoxic control; H/R, hypoxia/reoxygenation; siNC, negative-control siRNA; siCPT1A, CPT1A-targeting siRNA; TMRE, tetramethylrhodamine ethyl ester; LDH, lactate dehydrogenase. ns: not significant, * *p* < 0.05, ** *p* < 0.01, *** *p* < 0.001.

To further examine the functional involvement of the PPARα/CPT1A axis in proximal tubular cell injury, HK-2 cells were subjected to hypoxia/reoxygenation (H/R) and treated with a PPARα agonist, with or without CPT1A knockdown. Compared with control cells, H/R markedly reduced the protein levels of PPARα, CPT1A, ATP5A, and SLC34A1, while increasing cleaved PARP1, consistent with impaired metabolic and transport programs and enhanced cellular injury (Figure 9A). Pharmacological activation of PPARα substantially restored CPT1A, ATP5A, and SLC34A1 expression and reduced cleaved PARP1 levels under H/R conditions. In contrast, CPT1A knock-down significantly attenuated these protective effects. Notably, CPT1A depletion did not significantly alter PPARα protein abundance in PPARα agonist-treated cells, whereas the recovery of ATP5A and SLC34A1 was partially reversed and cleaved PARP1 was increased. These findings support CPT1A as an important downstream contributor to the protective effects associated with PPARα activation. Consistent with the immunoblotting results, TMRE staining showed a marked loss of mitochondrial membrane potential following H/R, as indicated by reduced TMRE fluorescence intensity. PPARα agonist treatment significantly restored TMRE fluorescence, whereas CPT1A knockdown diminished this recovery (Figure 9B,C). Similarly, H/R reduced cellular ATP levels, which were partially restored by PPARα activation and subsequently decreased following CPT1A knockdown (Figure 9D). Conversely, LDH release was markedly increased after H/R, reduced by PPARα agonist treatment, and increased again after CPT1A depletion (Figure 9E). Together, these results indicate that pharmacological activation of PPARα partially preserves mitochondrial function, cellular energy status, and the proximal tubular phenotype while limiting H/R-induced cellular injury and that these protective effects are at least partly dependent on CPT1A.

## 4. Discussion

In this study, temporal single-nucleus transcriptomics defined time-associated metabolic and cell-state remodeling of the proximal tubule during early ischemic AKI, complemented by in vivo and in vitro experimental assessments of selected molecular and functional findings. The central finding was that suppression of the PPARα/CPT1A-associated fatty acid oxidation program was most evident at 12 h and coincided with reduced mitochondrial and transport programs, increased injury-associated transcription, and expansion of stress-, injury-, repair-, and cycling-associated PT states. Several metabolic and transport features showed partial restoration at 2 d, although considerable heterogeneity persisted among biological samples and PT populations. Exploratory communication analysis further suggested that these epithelial changes occurred within a broader reorganization of the renal signaling environment. Together, these results extend previous single-cell studies of kidney injury by linking temporal metabolic changes to PT-state remodeling and by supporting the principal transcriptomic patterns at the protein, tissue, and cellular levels.

The temporal design showed that metabolic dysfunction did not develop uniformly after ischemic injury. At 4 h, the fatty acid oxidation score was relatively preserved, whereas oxidative phosphorylation and proximal tubular transport programs were already reduced. This pattern may reflect an early adaptive response among surviving tubular cells, although transcriptional module scores cannot establish increased fatty acid oxidation flux. By 12 h, the fatty acid oxidation score and the expression of *Ppara*, *Cpt1a*, *Sdhb*, and *Slc34a1* reached their lowest levels, while the injury program was most prominent. This time point therefore represented the clearest convergence of metabolic suppression, mitochondrial disturbance, loss of transport identity, and epithelial injury. At 2 d, several metabolic and transport programs moved toward the Sham level, yet injury-, repair-, and cycling-associated populations remained abundant. Recovery of sample-averaged gene expression can therefore coexist with persistent dysfunction in specific cell states. This distinction is important because improvement in bulk or pseudobulk expression may overestimate epithelial normalization when injured and incompletely differentiated populations remain present. Importantly, these temporal kinetics were derived from the GSE139107 transcriptomic series. The independent animal experiment was performed only at 24 h after reperfusion and therefore provides cross-sectional support for selected molecular changes rather than direct experimental validation of the 4 h–12 h–2 d temporal profile.

The early enrichment of the PT_S3 state is consistent with the recognized vulnerability of the S3 segment to ischemic stress. This segment is located mainly in the outer stripe of the outer medulla, where oxygen availability is limited despite high transport-related energy demand. In the present analysis, PT_S3 predominance at 4 h may represent an early segment-associated response to reduced oxygen delivery and mitochondrial stress. At 12 h, repair-associated PT cells became the dominant annotated state. This designation reflects activation of epithelial stress, dedifferentiation, proliferation, and repair-related transcriptional programs and does not imply completed regeneration. The persistence of injury markers within this compartment suggests that these cells occupied an intermediate state between acute damage and potential redifferentiation. The emergence of cycling PT cells and the marked inter-animal variability at 2 d further indicate that individual kidneys entered early repair with different proportions of surviving, activated, proliferating, and persistently injured cells. Previous single-nucleus and lineage-tracing studies have similarly identified heterogeneous adaptive, proliferative, inflammatory, and failed-repair PT states with distinct temporal distributions and long-term outcomes [26,27,28,29].

Trajectory inference provided a complementary framework for organizing this heterogeneity. Monocle3 placed mature PT cells near the trajectory root and computationally ordered stress-, repair-, cycling-, and injury-associated states toward later pseudotime positions. The accompanying decline in fatty acid oxidation and transport scores, together with increased injury signaling, indicated that loss of metabolic competence and differentiated identity occurred along the inferred transcriptional continuum. Slingshot produced a broadly concordant state ordering, supporting the robustness of the overall topology across analytical approaches. These findings require cautious interpretation. Pseudotime represents a computational ordering of transcriptionally related cells and does not show that every mature PT cell passes through each annotated state. Root selection, dimensional reduction, cluster annotation, and cell abundance can influence the resulting trajectory. In addition, Monocle3 and Slingshot were applied to the same cells and low-dimensional representation. Their agreement therefore supports computational robustness but does not constitute independent biological validation. Longitudinal single-cell profiling combined with lineage tracing will be required to define the origins and eventual outcomes of the identified PT states.

The coordinated reduction in PPARα and CPT1A is particularly relevant to proximal tubular metabolic fitness. Mature PT cells depend heavily on mitochondrial fatty acid oxidation to generate ATP for sodium-dependent solute transport and maintenance of brush-border polarity [30,31,32]. PPARα regulates genes involved in fatty acid uptake, β-oxidation, and mitochondrial metabolism, whereas CPT1A controls the entry of long-chain fatty acids into mitochondria. Experimental preservation of proximal tubular PPARα activity supports fatty acid oxidation, mitochondrial gene expression, renal morphology, and kidney function after ischemic or nephrotoxic injury. Conversely, defective fatty acid oxidation promotes lipid accumulation, energetic insufficiency, tubular dedifferentiation, and kidney damage. PGC-1α-dependent NAD biosynthesis and mitochondrial recovery also contribute to renal resistance and ischemic stress. The current findings add temporal and cell-state resolution to this framework by showing that PPARα/CPT1A-associated suppression was most apparent during peak injury and was linked to PT states with reduced transport identity.

The experimental findings provided complementary evidence for selected molecular features identified in the transcriptomic analysis. The mouse ischemia–reperfusion experiments were performed at a single 24-h endpoint and therefore represent cross-sectional protein, tissue, and functional assessments rather than direct validation of the transcriptomic temporal kinetics. In the ischemia–reperfusion model, reductions in renal PPARα and CPT1A occurred together with impaired kidney function, histological tubular damage, and induction of KIM-1 and NGAL. Hypoxia–reoxygenation similarly reduced PPARα and CPT1A and increased cleaved caspase-3 in tubular epithelial cells. ATP5A and SDHB were also reduced in injured kidneys, supporting impairment of mitochondrial oxidative metabolism. The relatively stable *Atp5a1* transcript level despite lower ATP5A protein abundance suggests that transcriptomic measurements alone may not fully capture mitochondrial dysfunction. Potential contributors include post-transcriptional regulation, protein degradation, and loss of mitochondrial mass. In parallel, reduced SLC34A1 expression in pseudobulk profiles, kidney lysates, and LTL-positive tubules indicated disruption of differentiated PT transport identity. Collectively, these findings support a close relationship among metabolic competence, mitochondrial integrity, and maintenance of the mature PT phenotype. Importantly, the newly added functional intervention experiments further strengthened this relationship. Pharmacological activation of PPARα partially restored CPT1A, ATP5A, and SLC34A1 expression, improved mitochondrial membrane potential and ATP production, and reduced cellular injury under H/R conditions. CPT1A knockdown attenuated these protective effects, supporting a CPT1A-dependent component of PPARα agonist-associated protection. These findings provide functional evidence for involvement of the PPARα/CPT1A axis in the HK-2 H/R model, although they do not establish that all temporal transcriptomic alterations observed in vivo are mediated by this pathway. An additional limitation concerns the use of HK-2 cells as the in vitro proximal tubular model. Although HK-2 cells are widely used to investigate renal tubular responses to hypoxia/reoxygenation, they are an immortalized cell line and may not fully retain the metabolic, mitochondrial, transport, and differentiation characteristics of primary human proximal tubular epithelial cells. Immortalization and adaptation to prolonged in vitro culture may alter substrate utilization, bioenergetic state, mitochondrial function, and expression of differentiated tubular markers. Accordingly, the H/R and PPARα agonist/CPT1A-knockdown experiments should be interpreted as model-specific functional evidence rather than as direct reproduction of primary human PT physiology. The concordant in vivo findings provide an additional level of support but do not eliminate this limitation. Future validation in primary human proximal tubular epithelial cells, kidney organoids, or other physiologically relevant human renal models will be required to determine the generalizability of these findings.

Exploratory CellChat analysis suggested that PT remodeling occurred within a broader reorganization of the renal signaling environment. The pooled early-AKI analysis was retained as a descriptive overview, while additional time-specific CellChat models were constructed separately for Sham, 4 h, 12 h, and 2 d to resolve temporal communication dynamics. These analyses revealed both persistent and time-dependent predicted communication patterns. In particular, PT-centered Nrg1–Erbb4 interactions involving thick ascending limb populations were repeatedly detected from 4 h through 2 d, suggesting a sustained candidate relationship rather than a 12 h-specific event. In contrast, the reproducibility of several other pathway-level predictions varied across time points and biological kidneys, indicating that communication remodeling during early AKI is temporally heterogeneous. Individual-kidney support analyses further showed that pooled network scores alone do not establish reproducible biological changes across animals. These findings should therefore be regarded as hypothesis-generating. CellChat infers communication from transcript abundance and curated ligand-receptor relationships and cannot demonstrate ligand secretion, receptor activation, downstream signaling, or spatial contact. Spatial localization, protein-level assessment, and pathway perturbation will be required to establish the biological relevance of these predicted interactions. The state heterogeneity observed in this mouse dataset may be relevant to human AKI. Single-cell analysis of human kidney tissue has identified recurrent injury-associated epithelial states characterized by hypoxia, oxidative stress, inflammatory signaling, and epithelial plasticity, together with substantial inter-individual variation. Urinary single-cell sequencing has also captured tubular injury and repair-related populations in patients with AKI [33], indicating that epithelial-state dynamics may be accessible through minimally invasive sampling. These studies support the broader relevance of state-based analysis, although they do not establish that the specific PPARα/CPT1A temporal pattern identified here is conserved in clinical disease. Human AKI is influenced by age, comorbidities, medications, sepsis, nephrotoxins, and variable durations of ischemia. Validation in human kidney tissue, urine-derived tubular cells, or longitudinal clinical cohorts will therefore be necessary before the proposed metabolic framework can inform biomarker development or therapeutic stratification.

Several strengths of this study should be noted. The transcriptomic dataset contained three independent kidneys at each of four early time points, and condition-level statistical analyses used the kidney as the unit of replication. Sample-level pseudobulk analysis reduced the risk of treating individual nuclei as independent observations. Whole-kidney profiling established the multicellular context, while PT subclustering resolved segment-specific and injury-related states. Two trajectory methods were used to examine computational robustness, and the main transcriptional patterns were evaluated in animal and cell models. The study also has important limitations. The transcriptomic analysis relied on one public mouse dataset with limited biological replication and lacked an independent transcriptomic cohort. The availability of only three biological kidneys per time point limits statistical precision and power, particularly for modest transcriptional effects, and prevents robust empirical assessment of normality and variance assumptions. Empirical Bayes moderation improves variance estimation across genes but does not increase the number of independent biological replicates. Accordingly, FDR control remains conditional on the statistical model and predefined testing family and should not be interpreted as eliminating uncertainty for individual findings. We therefore report effect sizes, confidence intervals, exact numerical *p* values, kidney-level observations, and multiple sensitivity analyses alongside adjusted *p* values. Total-PT pseudobulk profiles may reflect both changes within PT states and changes in state abundance. Module scores do not directly measure fatty acid oxidation flux, mitochondrial respiration, ATP production, or solute transport. Because some genes used for PT-state annotation also contribute to functional scoring, state-level module differences are potentially circular and are therefore interpreted descriptively. The annotation-marker-exclusion sensitivity analyses reduce dependence on the declared marker set but do not make the resulting analyses biologically independent, because both annotation and functional scoring derive from the same transcriptomic dataset. Trajectory inference cannot establish lineage relationships. Although time-specific CellChat models and individual-kidney support analyses were added to improve temporal resolution and assessment of reproducibility, these analyses remain computational predictions derived from the same transcriptomic dataset. The kidney-level support analysis is descriptive and does not constitute a formal between-condition differential-communication test. Functional perturbation of PPARα and CPT1A was performed in the HK-2 H/R model, but equivalent causal intervention has not yet been performed in vivo. An additional limitation is that the in vivo validation experiments were conducted exclusively in male mice. Sex is an important biological variable in renal ischemia–reperfusion injury and metabolic regulation; therefore, the present animal experiments do not address potential sex-dependent differences in injury severity, mitochondrial responses, or PPARα/CPT1A-associated metabolic remodeling. The in vivo findings should consequently be interpreted as being specific to male physiology and should not be assumed to generalize to female animals. Future studies specifically designed to include both sexes and adequately powered for sex-by-injury or sex-by-intervention analyses will be required to determine the extent to which the observed responses are conserved across sexes.

Future studies should test whether restoration of PPARα/CPT1A activity preserves fatty acid oxidation, mitochondrial respiration, and differentiated PT identity after ischemic injury. Future studies should extend the present PPARα activation/CPT1A-depletion findings to in vivo models and determine whether preservation of this axis alters long-term proximal tubular repair and renal functional recovery. Longitudinal single-cell and spatial analyses, together with lineage tracing, will be needed to determine whether metabolic intervention changes the balance among adaptive repair, persistent injury, proliferation, and maladaptive remodeling. Overall, the present findings define an early ischemic AKI program in which suppression of PPARα/CPT1A-associated metabolism coincides with mitochondrial impairment, loss of mature transport identity, and remodeling of proximal tubular cell states. This framework provides a rationale for testing whether preservation of proximal tubular metabolic competence can promote adaptive kidney repair.

## Data Availability

The single-nucleus RNA sequencing dataset analyzed in this study is publicly available in the NCBI Gene Expression Omnibus under accession number GSE139107. Additional experimental data are available from the corresponding authors upon request.

## Supplementary Materials

The following supporting information can be downloaded at https://www.mdpi.com/article/10.3390/biomedicines14102317/s1, Figure S1: Whole-kidney sample distributions and composition changes during early ischemic AKI; Figure S2: Proximal tubular sample heterogeneity and composition across the early injury course; Figure S3: Kidney-level functional module-score comparisons during early ischemic AKI; Figure S4: Individual-kidney expression profiles of selected metabolic, mitochondrial, transport, and injury-associated genes; Figure S5: Sample-level and within-PT-subgroup sensitivity analyses; Figure S6: Gene-set enrichment analyses with full and annotation-marker-excluded rankings; Figure S7: Trajectory coverage and sensitivity analyses for proximal tubular pseudotime; Figure S8: Time-specific CellChat predictions and expression support for PT-centered communication; Data S1: Master_Statistics.xlsx; Data S2: All_gene_pseudobulk.xlsx; Data S3: Composition_and_annotation.xlsx; Data S4: Module_scores.xlsx; Data S5: Within_PT_and_sensitivity.xlsx; Data S6: GSEA.xlsx; Data S7: Trajectory.xlsx; Data S8: CellChat_input_audit.xlsx; Data S9: CellChat_networks.xlsx; Data S10: CellChat_kidney_support.xlsx; Data S11: Nrg1_Erbb4_expression.xlsx; Table S1: Mapping of original technical sequencing libraries to reconstructed biological kidney samples in GSE139107; Table S2: Antibodies and lectin detection reagents used for experimental validation.
