# Supplementary material for: Temporal Single-Nucleus Transcriptomics Defines PPARα/CPT1A-Associated Metabolic Reprogramming During Proximal Tubular Injury and Repair
Source: Biomedicines. 2026 Oct 12;14(10):2317. doi: 10.3390/biomedicines14102317 (PMC13701615; doi:10.3390/biomedicines14102317)
Supplement: Supplementary file 1 [file biomedicines-14-02317-s001.zip › Supplementary Figures.pdf]

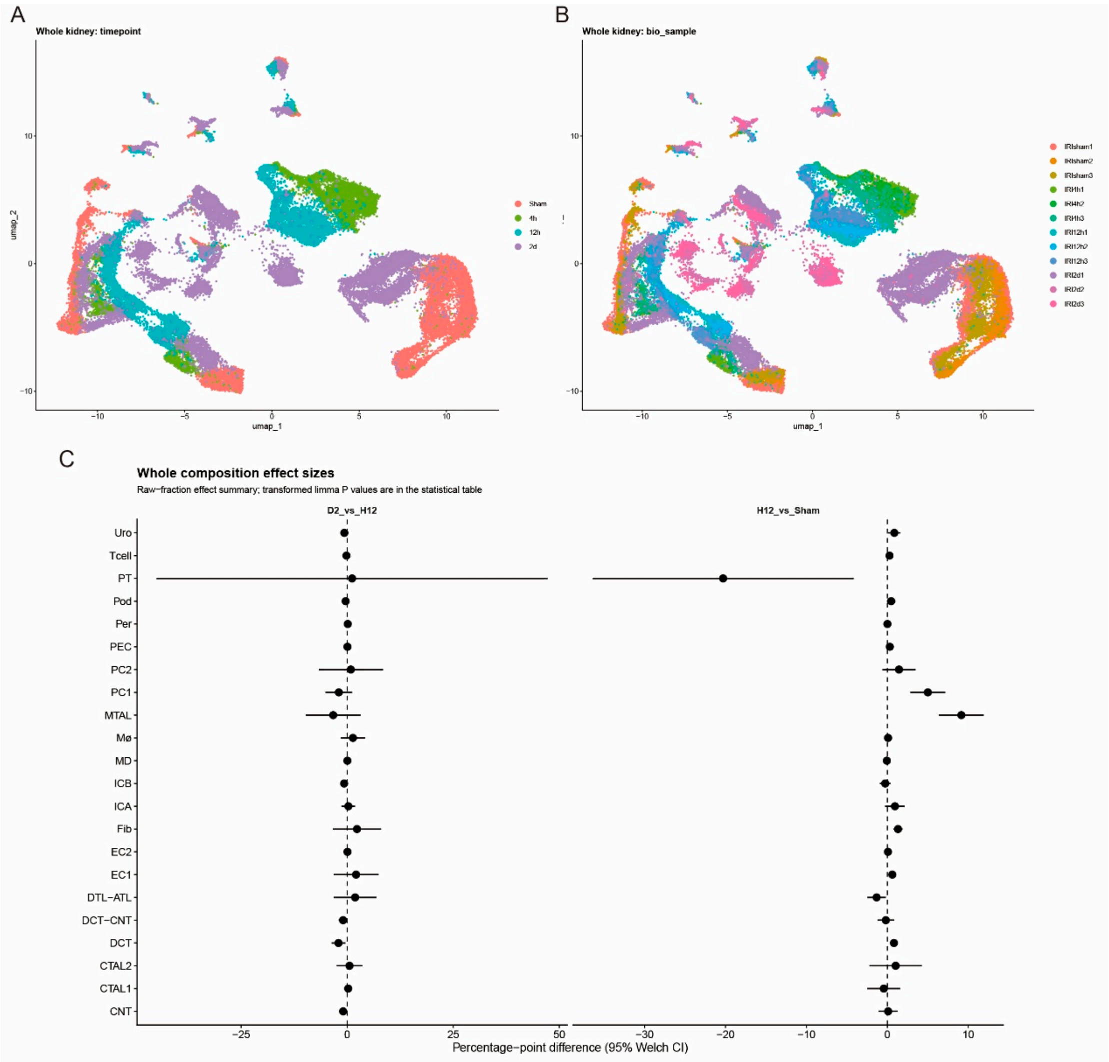

Supplementary Figure S1. Whole-kidney sample distributions and composition changes during early ischemic AKI. (A) UMAP representation of whole-kidney nuclei colored by experimental time point (Sham, 4 h, 12 h, and 2 d).(B) UMAP representation of the same nuclei colored by biological kidney, showing the contributions of the 12 independent samples to the whole-kidney embedding.(C) Effect-size summaries for selected whole-kidney cell-population proportions across temporal contrasts. Points indicate estimated percentage-point differences in cell-type abundance, and horizontal lines indicate corresponding 95% Welch confidence intervals. Dashed vertical lines indicate no change. Raw-fraction effect estimates are shown for descriptive interpretation; inferential composition statistics based on the prespecified transformed-scale model are provided in Supplementary Data S3. Each biological kidney was treated as one independent replicate.

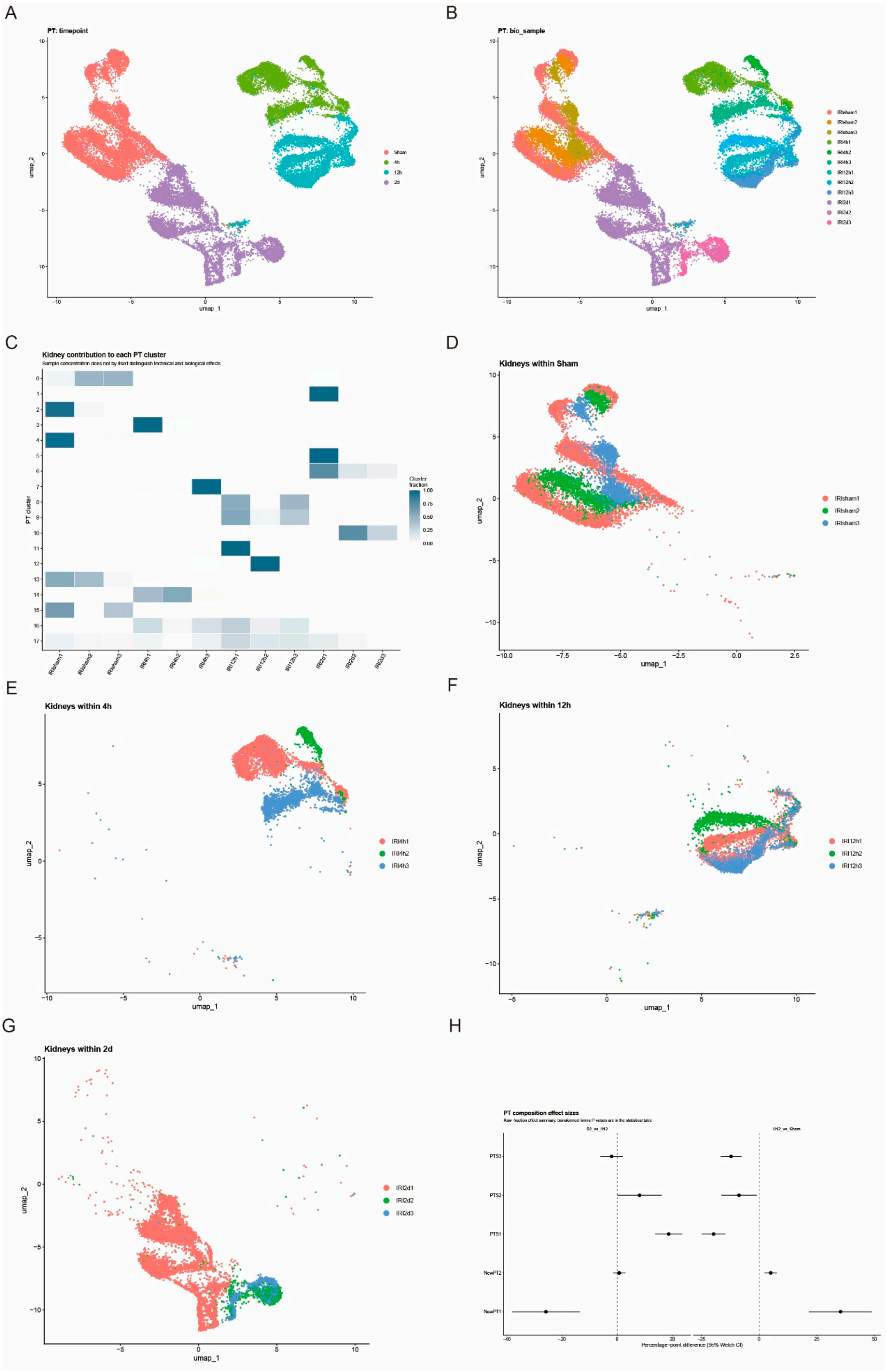

Supplementary Figure S2. Proximal tubular sample heterogeneity and composition across the early injury course. (A) UMAP representation of proximal tubular nuclei colored by

experimental time point. (B) UMAP representation of proximal tubular nuclei colored by biological kidney.(C) Heatmap showing the contribution of individual biological kidneys to each numerical PT cluster. Values represent the fraction of cells within each cluster contributed by the corresponding kidney and are used to visualize sample concentration and heterogeneity. (D–G) UMAP distributions of proximal tubular nuclei from the three independent kidneys within Sham (D), 4 h (E), 12 h (F), and 2 d (G), respectively. (H) Effect-size summaries for selected PT subgroup composition contrasts. Points indicate estimated percentage-point differences in subgroup abundance, and horizontal lines indicate 95% Welch confidence intervals. Dashed vertical lines indicate no change. These analyses describe sample and subgroup heterogeneity and do not independently validate PT-state annotations.

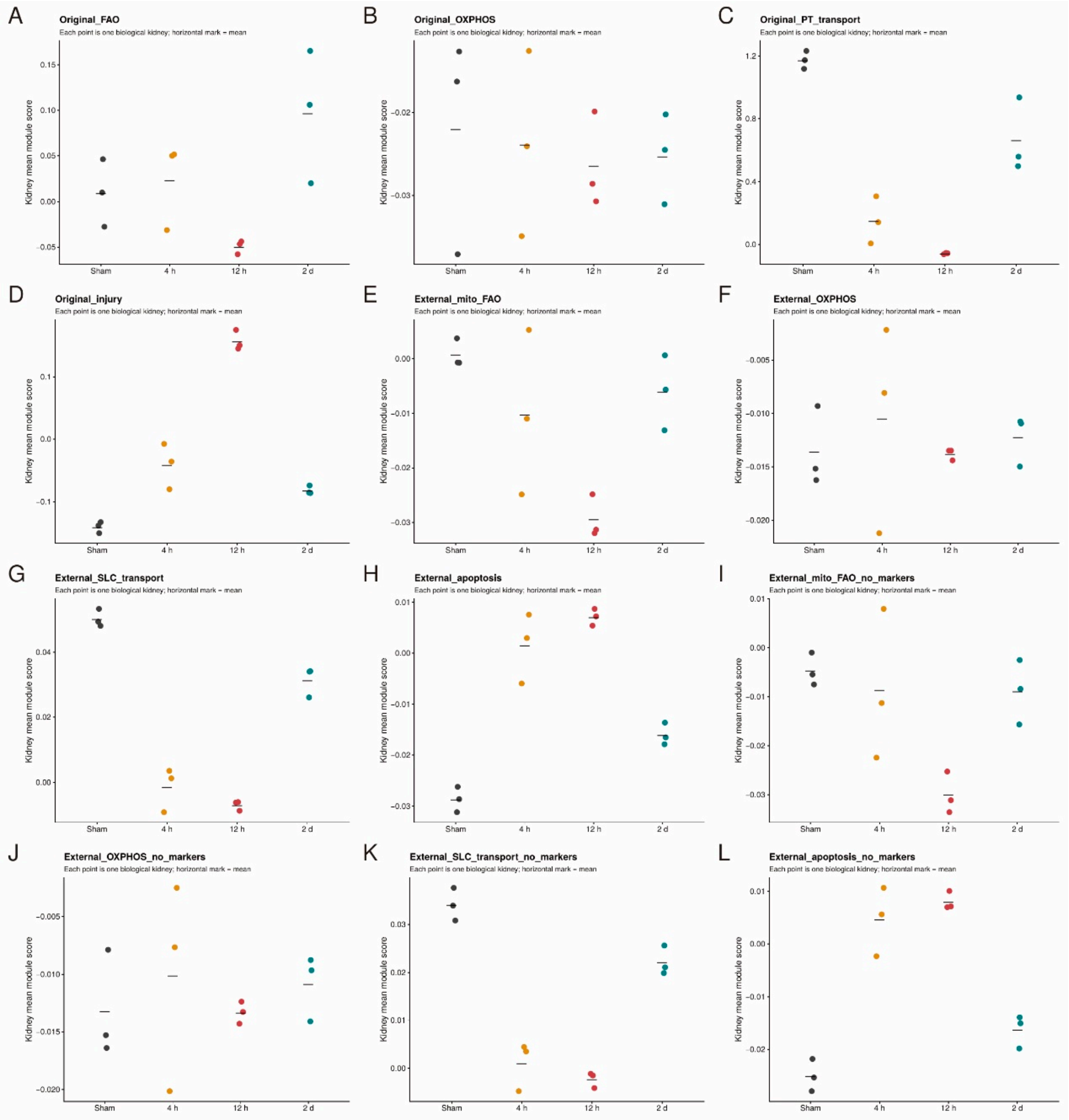

Supplementary Figure S3. Kidney-level functional module-score comparisons during early ischemic AKI. (A–D) Kidney-level scores for the original fatty acid oxidation (A), oxidative phosphorylation (B), proximal tubular transport (C), and injury (D) gene modules.

(E–H) Kidney-level scores derived from external mitochondrial fatty acid oxidation (E), oxidative phosphorylation (F), SLC-mediated transport (G), and apoptosis (H) gene sets. (I–L) Corresponding external mitochondrial fatty acid oxidation (I), oxidative phosphorylation (J), SLC-mediated transport (K), and apoptosis (L) scores recalculated after exclusion of the predefined annotation/review genes. Each point represents one independent biological kidney, and the horizontal mark indicates the group mean. Marker-excluded analyses were performed as sensitivity analyses to reduce dependence on genes used during PT-state annotation and are not interpreted as independent biological validation. Complete gene-set definitions, kidney-level values, effect estimates, confidence intervals, and statistical results are provided in Supplementary Data S4.

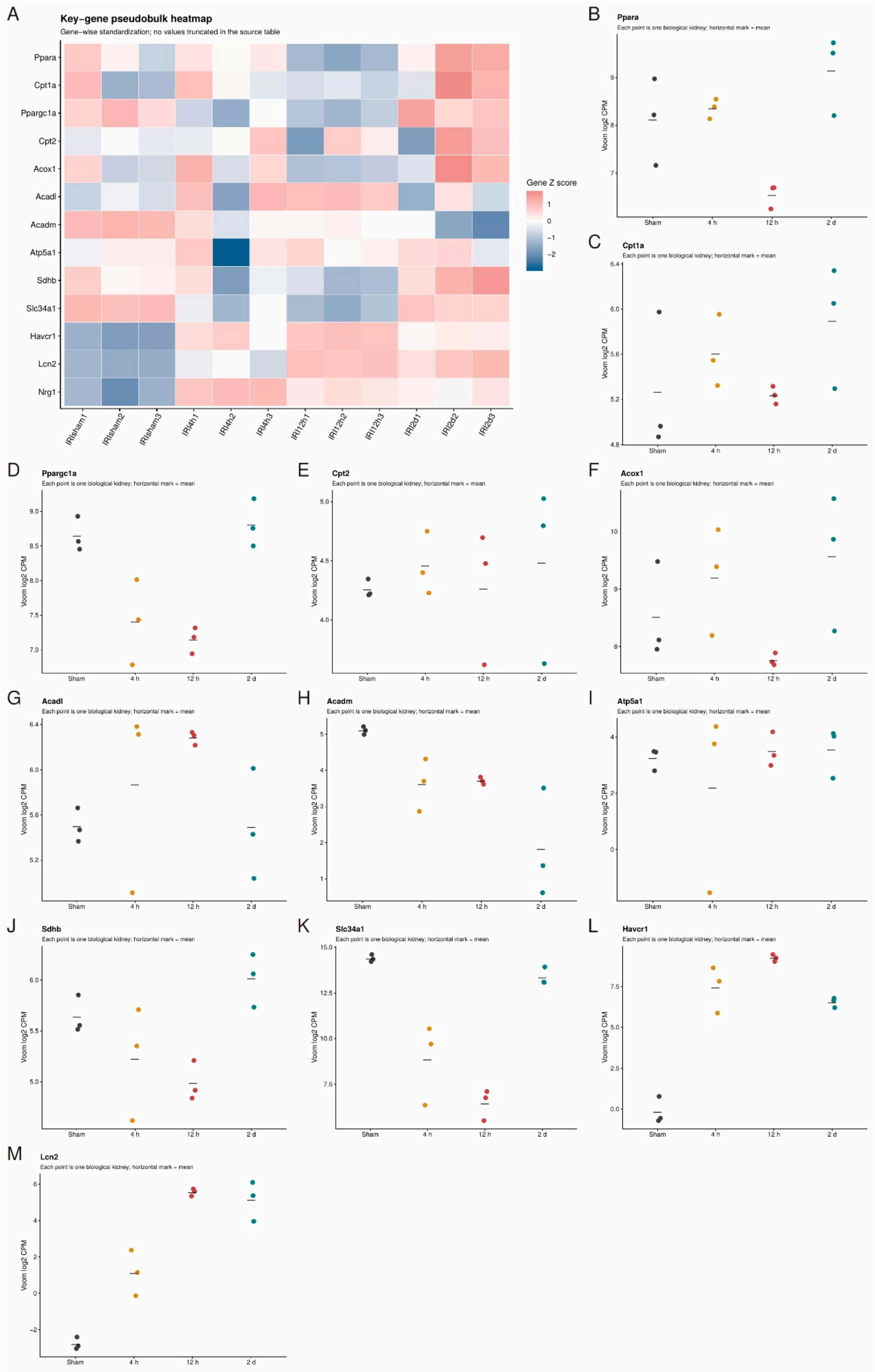

Supplementary Figure S4. Individual-kidney expression profiles of selected metabolic, mitochondrial, transport, and injury-associated genes. (A) Heatmap showing gene-wise

standardized pseudobulk expression of selected transcripts across the 12 biological kidneys. Values were standardized separately for each gene to visualize relative expression patterns across samples. (B–M) Kidney-level voom log2 counts-per-million (CPM) expression of *Ppara* (B), *Cpt1a* (C), *Ppargc1a* (D), *Cpt2* (E), *Acox1* (F), *Acadl* (G), *Acadm* (H), *Atp5a1* (I), *Sdhb* (J), *Slc34a1* (K), *Havcr1* (L), and *Lcn2* (M) across Sham, 4 h, 12 h, and 2 d. Each point represents one independent biological kidney, and the horizontal mark indicates the group mean. Formal differential-expression inference was performed using the kidney-level pseudobulk model rather than visual separation of individual points.

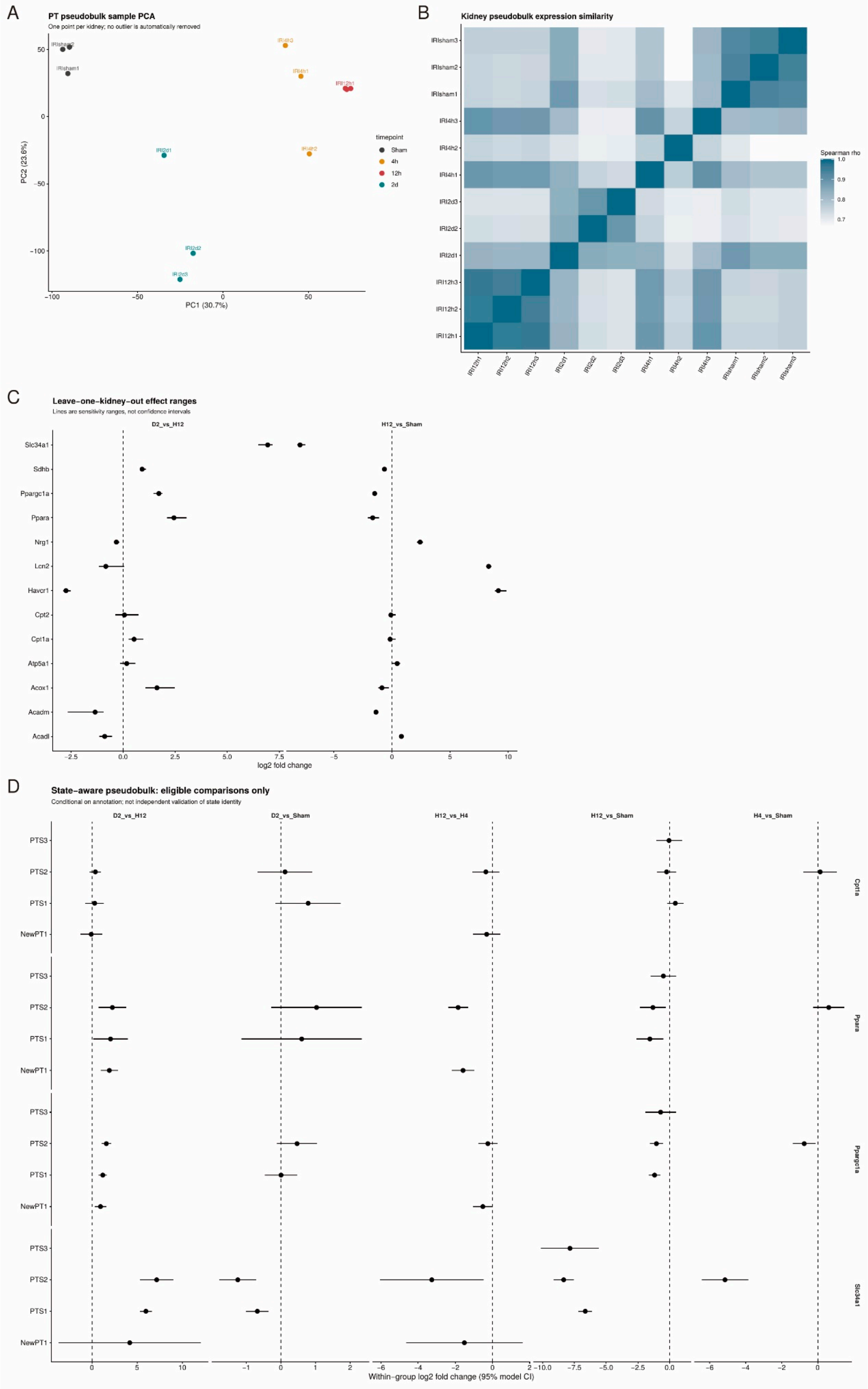

Supplementary Figure S5. Sample-level and within-PT-subgroup sensitivity analyses. (A)

Principal-component analysis of kidney-level PT pseudobulk expression profiles. Each point represents one biological kidney and is colored by experimental time point. No sample was automatically excluded on the basis of its position in the PCA space. (B) Heatmap of pairwise Spearman correlations among kidney-level PT pseudobulk expression profiles. (C) Systematic leave-one-kidney-out sensitivity analysis for selected genes in the 12 h versus Sham and 2 d versus 12 h contrasts. Points indicate the full-cohort log2 fold-change estimate, and horizontal lines indicate the range of estimates obtained after sequential omission of each eligible kidney. These ranges represent sensitivity ranges and are not confidence intervals. (D) Within-published-PT-subgroup pseudobulk estimates for selected genes and eligible temporal contrasts. Points indicate subgroup-specific log2 fold changes and horizontal lines indicate 95% model confidence intervals. Only subgroup-by-comparison combinations meeting the predefined biological-replication and cell-number criteria were analyzed. These analyses are conditional on the underlying annotation and are presented as sensitivity analyses rather than independent validation of PT-state identity.

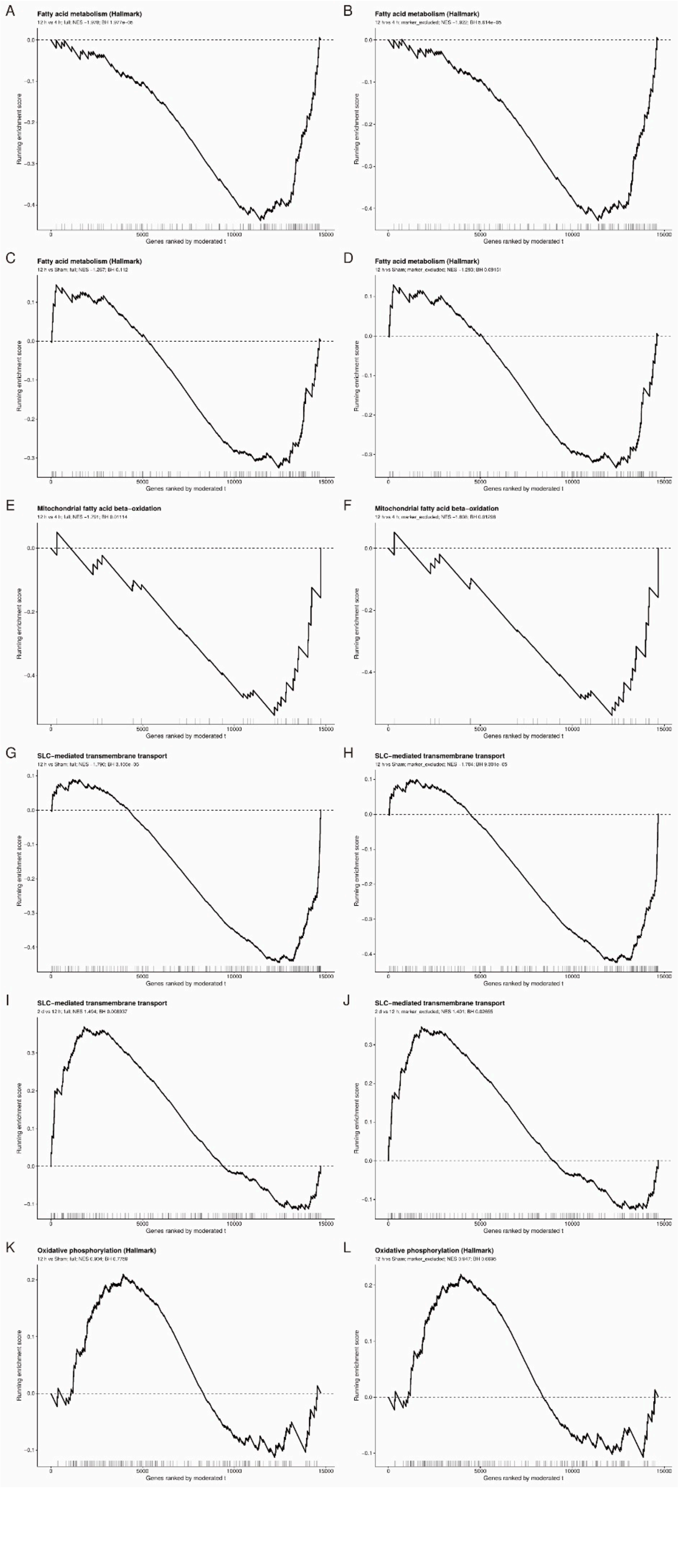
A
Fatty acid metabolism (Hallmark)
12 h vs 4 h; full; NES −1.978; BH 1.977e−05
B
Fatty acid metabolism (Hallmark)
12 h vs 4 h; marker_excluded; NES −1.922; BH 5.614e−05
C
Fatty acid metabolism (Hallmark)
12 h vs Sham; full; NES −1.267; BH 0.112
D
Fatty acid metabolism (Hallmark)
12 h vs Sham; marker_excluded; NES −1.293; BH 0.09151
E
Mitochondrial fatty acid beta−oxidation
12 h vs 4 h; full; NES −1.791; BH 0.01114
F
Mitochondrial fatty acid beta−oxidation
12 h vs 4 h; marker_excluded; NES −1.808; BH 0.01298
G
SLC−mediated transmembrane transport
12 h vs Sham; full; NES −1.790; BH 3.100e−05
H
SLC−mediated transmembrane transport
12 h vs Sham; marker_excluded; NES −1.704; BH 9.301e−05
I
SLC−mediated transmembrane transport
2 d vs 12 h; full; NES 1.494; BH 0.008937
J
SLC−mediated transmembrane transport
2 d vs 12 h; marker_excluded; NES 1.401; BH 0.02655
K
Oxidative phosphorylation (Hallmark)
12 h vs Sham; full; NES 0.904; BH 0.7759
L
Oxidative phosphorylation (Hallmark)
12 h vs Sham; marker_excluded; NES 0.947; BH 0.6695
Running enrichment score
Genes ranked by moderated t

Supplementary Figure S6. Gene-set enrichment analyses with full and annotation-marker-excluded rankings. (A, B) Hallmark fatty acid metabolism enrichment for 12 h versus 4 h using the full gene ranking (A) and the corresponding annotation-marker-excluded ranking (B). (C, D) Hallmark fatty acid metabolism enrichment for 12 h versus Sham using the full (C) and marker-excluded (D) rankings. (E, F) Mitochondrial fatty acid β-oxidation enrichment for 12 h versus 4 h using the full (E) and marker-excluded (F) rankings. (G, H) SLC-mediated transmembrane transport enrichment for 12 h versus Sham using the full (G) and marker-excluded (H) rankings. (I, J) SLC-mediated transmembrane transport enrichment for 2 d versus 12 h using the full (I) and marker-excluded (J) rankings. (K, L) Hallmark oxidative phosphorylation enrichment for 12 h versus Sham using the full (K) and marker-excluded (L) rankings. Genes were ranked according to the moderated test statistic from the kidney-level pseudobulk analysis. Running enrichment scores are shown along the ranked gene list, and the normalized enrichment score (NES) and Benjamini–Hochberg-adjusted P value are indicated in each panel. Marker-excluded analyses were used as robustness analyses to assess the influence of annotation-gene overlap. Complete enrichment results are provided in Supplementary Data S6.

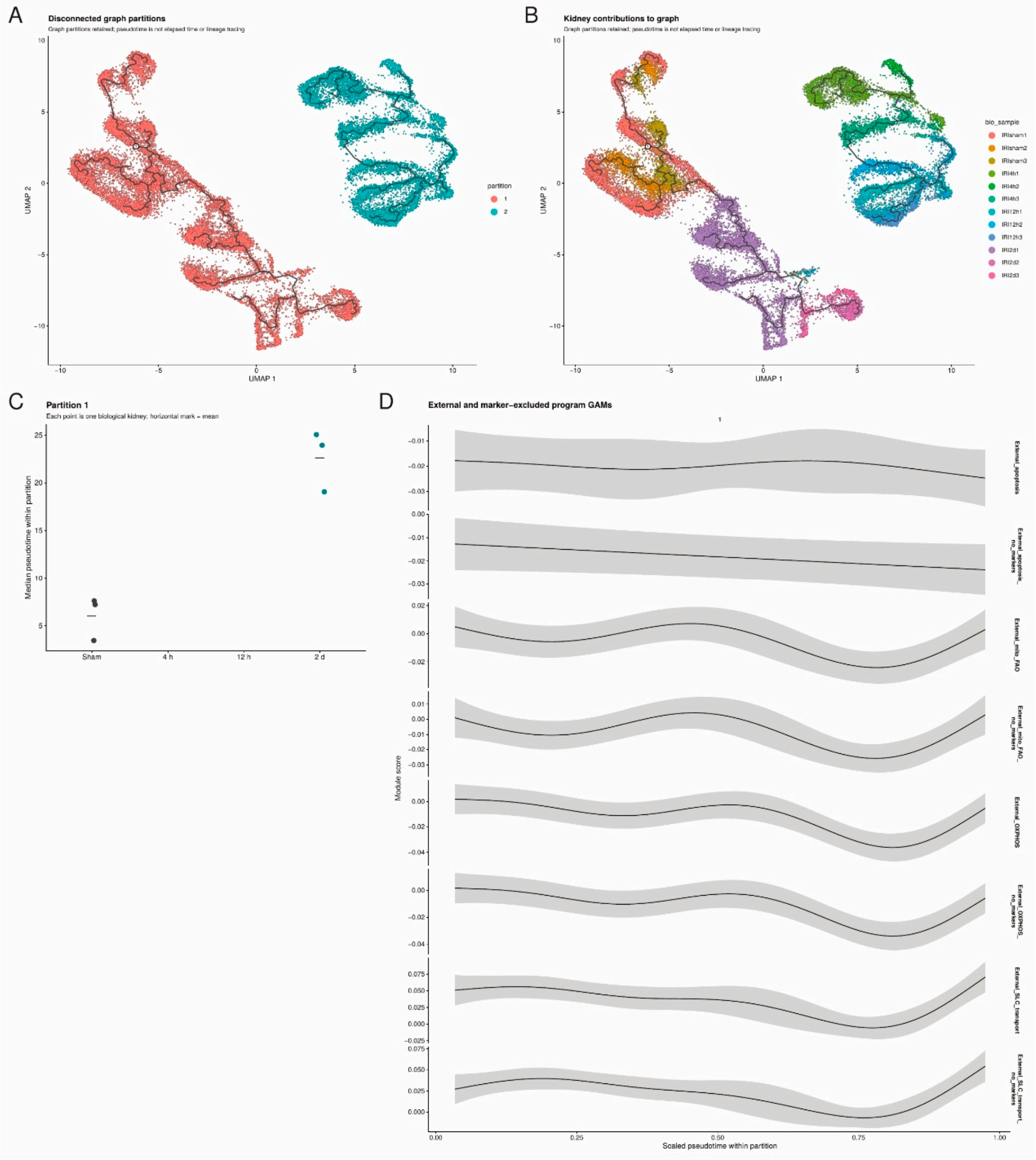

Supplementary Figure S7. Trajectory coverage and sensitivity analyses for proximal tubular pseudotime. (A) Monocle3 graph partitions projected onto the PT UMAP, demonstrating two disconnected graph partitions. (B) Contribution of individual biological kidneys to the inferred graph partitions. (C) Median pseudotime within the rooted partition for eligible biological kidneys. Each point represents one kidney, and the horizontal mark indicates the group mean. Time points without sufficient finite-pseudotime coverage within the rooted partition were not assigned kidney-level median values for inferential comparison. (D) Conditional generalized additive model fits for external and annotation-marker-excluded functional programs along scaled pseudotime within the estimable rooted partition. Lines indicate fitted trends and shaded regions indicate pointwise 95% confidence intervals. These analyses are conditional on the available rooted partition and do not establish lineage tracing or a continuous trajectory connecting all four experimental time points.

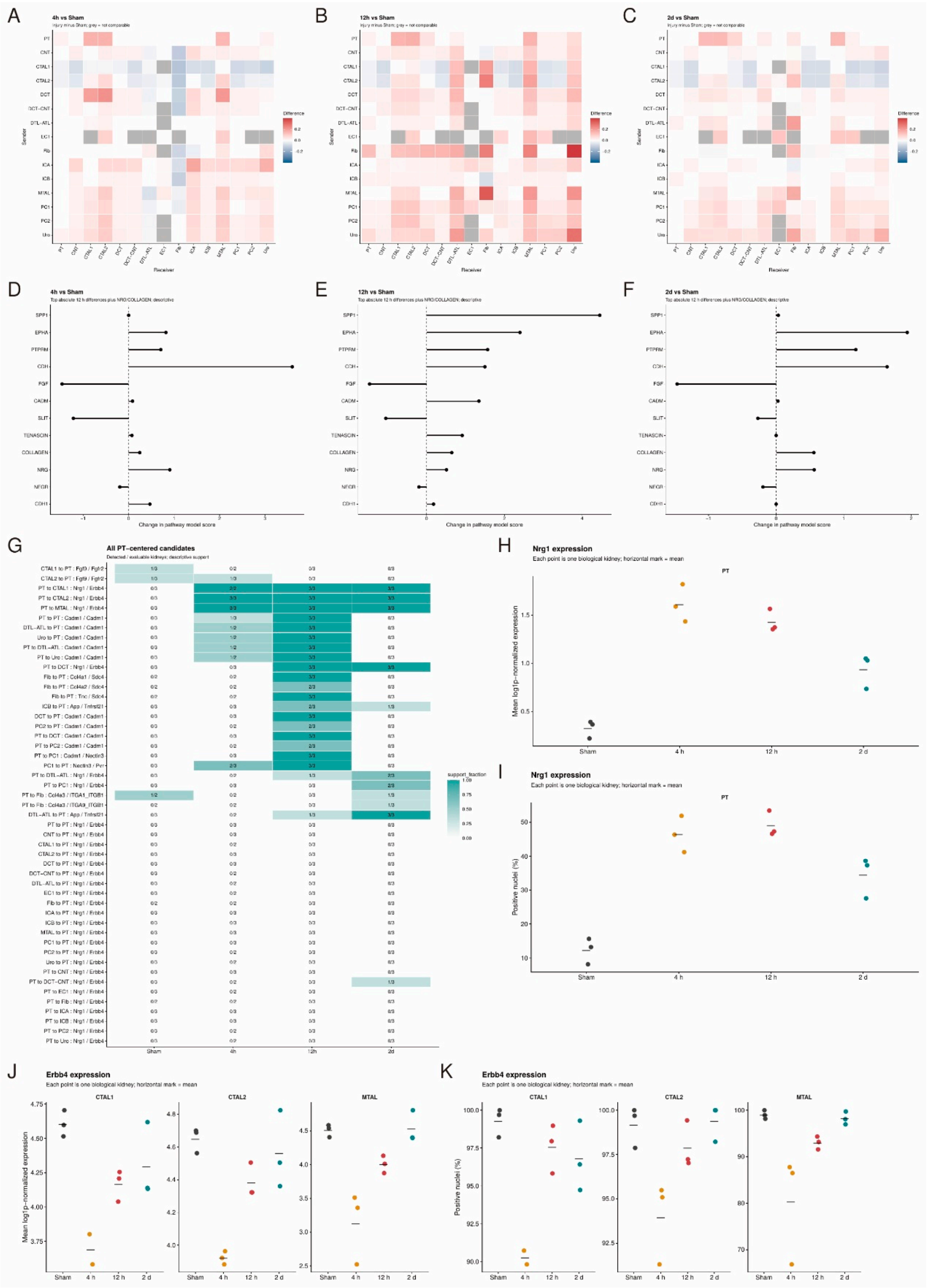

Supplementary Figure S8. Time-specific CellChat predictions and expression support for PT-centered communication. (A–C) Differences in predicted sender-receiver communication strength for 4 h versus Sham (A), 12 h versus Sham (B), and 2 d versus Sham (C). Positive values indicate greater predicted communication in the injury condition relative to Sham, whereas negative values indicate lower predicted communication. Gray cells denote sender-receiver combinations that were not comparable under the predefined eligibility criteria. (D–F)

Changes in pathway-level CellChat model scores for 4 h versus Sham (D), 12 h versus Sham (E), and 2 d versus Sham (F). The displayed pathways include the largest time-associated differences together with selected NRG- and COLLAGEN-related pathways and are interpreted descriptively. (G) Kidney-level support matrix for retained PT-centered ligand-receptor candidates across Sham, 4 h, 12 h, and 2 d. Each cell reports the number of kidneys in which the predicted interaction was detected relative to the number of evaluable kidneys, and the color scale represents the corresponding support fraction. (H, I) Kidney-level PT *Nrg1* expression shown as mean log1p-normalized expression (H) and percentage of *Nrg1*-positive nuclei (I). (J, K) Kidney-level *Erbb4* expression in CTAL1, CTAL2, and MTAL recipient populations shown as mean log1p-normalized expression (J) and percentage of *Erbb4*-positive nuclei (K). Each point in the expression panels represents one eligible biological kidney, and the horizontal mark indicates the group mean. CellChat outputs and kidney-support fractions are descriptive predictions and were not interpreted as formal biological-kidney between-condition significance tests. Predicted ligand-receptor relationships do not establish ligand secretion, receptor activation, spatial contact, or downstream functional signaling. Complete input audits, network summaries, kidney-support records, and *Nrg1/Erbb4* expression data are provided in Supplementary Data S8–S11.
