# Supplementary material for: Temporal Single-Nucleus Transcriptomics Defines PPARα/CPT1A-Associated Metabolic Reprogramming During Proximal Tubular Injury and Repair
Source: Biomedicines. 2026 Oct 12;14(10):2317. doi: 10.3390/biomedicines14102317 (PMC13701615; doi:10.3390/biomedicines14102317)
Supplement: Supplementary file 1 [file biomedicines-14-02317-s001.zip › Supplementary Tables.pdf]

Supplementary Table S1. Mapping of original technical sequencing libraries to reconstructed biological kidney samples in GSE139107.

Time point	Reconstructed biological sample ID	Original technical sequencing library ID(s)	Technical libraries, n	Retained nuclei, n
Sham	IRIsham1	IRIsham1b1; IRIsham1b2	2	14,766
Sham	IRIsham2	IRIsham2	1	5,400
Sham	IRIsham3	IRIsham3	1	5,095
4 h	IRI4h1	IRI4h1	1	6,631
4 h	IRI4h2	IRI4h2	1	1,846
4 h	IRI4h3	IRI4h3	1	5,272
12 h	IRI12h1	IRI12h1b1; IRI12h1b2	2	10,988
12 h	IRI12h2	IRI12h2	1	4,593
12 h	IRI12h3	IRI12h3	1	4,443
2 d	IRI2d1	IRI2d1b1; IRI2d1b2	2	14,055
2 d	IRI2d2	IRI2d2b1; IRI2d2b2	2	4,497
2 d	IRI2d3	IRI2d3	1	1,940

Supplementary Table S2. Antibodies and lectin detection reagents used for experimental validation.

Target or reagent	Supplier, catalog no., and RRID	Host/clone and species reactivity	Application and working dilution	Expected molecula r weight (kDa)
PPARα	Invitrogen, Thermo Fisher Scientific PA1-822A; RRID: AB_2165595	Rabbit polyclonal; mouse	WB; 1:1,000	~52
CPT1A	Invitrogen, Thermo Fisher Scientific PA5-106270; RRID: AB_2853947	Rabbit polyclonal; human, mouse, rat	WB; 1:1,000	~88

Target or reagent	Supplier, catalog no., and RRID	Host/clone and species reactivity	Application and working dilution	Expected molecular weight (kDa)
KIM-1/HAVCR1	Proteintech 30948-1-AP; RRID: AB_3669790	Rabbit polyclonal; mouse, rat	IHC-P; 1:200	N/A for IHC; ~70 by WB
NGAL/LCN2	Invitrogen, Thermo Fisher Scientific MA5-38280; RRID: AB_2898195	Mouse monoclonal, clone 1A1B2; human, mouse	WB, 1:1,000; IHC-P, 1:200	~25
ATP5A1	Invitrogen, Thermo Fisher Scientific 459240; RRID: AB_2532234	Mouse monoclonal, clone 7H10BD4F9; bovine, human, mouse, rat	WB; 1 µg/mL	~54
SDHB	Invitrogen, Thermo Fisher Scientific MA5-26936; RRID: AB_2725309	Mouse monoclonal, clone OTI3F5; human, mouse, rat	WB; 1:2,000	~30
SLC34A1/NaPi-IIa	Novus Biologicals, Bio-Techne NBP2-42216; RRID: AB_2801654	Mouse monoclonal, clone 10B1.3E9; human, mouse	WB, 1:500; IF, 1:50	68.9 predicted; ~80 glycosylated
Cleaved caspase-3 (Asp175)	Cell Signaling Technology 9664; RRID: AB_2070042	Rabbit monoclonal, clone 5A1E; human, mouse, rat, monkey	WB; 1:1,000	17 and 19
β-actin	Invitrogen, Thermo Fisher Scientific MA1-140; RRID: AB_2536844	Mouse monoclonal, clone 15G5A11/E2; human, mouse, rat, dog, non-human primate	WB; 1:5,000	~42
Lotus tetragonolobus lectin, fluorescein conjugate	Vector Laboratories FL-1321-2; RRID: AB_2336559	Fluorescein-conjugated lectin	IF; 10 µg/mL	N/A

Abbreviations: HRP, horseradish peroxidase; IF, immunofluorescence; IHC-P, immunohistochemistry on paraffin-embedded sections; N/A, not applicable; RRID, Research Resource Identifier; WB, western blotting.
